# HabitWalk: A micro‐randomized trial to understand and promote habit formation in physical activity

**DOI:** 10.1111/aphw.12605

**Published:** 2024-10-10

**Authors:** Dario Baretta, Noemi Gillmann, Robert Edgren, Jennifer Inauen

**Affiliations:** ^1^ Institute of Psychology University of Bern Bern Switzerland

**Keywords:** commitment, cue‐behavior repetition, habit formation, physical activity, prompts and cues, time series

## Abstract

Habit is a key psychological determinant for physical activity behavior change and maintenance. This study aims to deepen the understanding of habit formation in physical activity and identify promotion strategies. We examined the habit formation trajectory and its relationships with cue‐behavior repetition (a cue‐triggered 15‐minute brisk walk) and unconditional physical activity (daily steps). We also tested whether the behavior change techniques (BCTs) ‘commitment’ and ‘prompts and cues’ promote habit, cue‐behavior repetition, and daily steps within persons. This micro‐randomized trial included a 7‐day preparatory and a 105‐day experimental phase delivered via the HabitWalk app. Participants (*N* = 24) had a 50% probability of receiving each BCT daily, leading to four conditions. Habit strength was assessed daily using the Self‐Report Behavioral Automaticity Index, while cue‐behavior repetition and steps were measured via an activity tracker. Person‐specific growth functions indicated that habit strength trajectories were highly idiosyncratic. Multilevel models indicated a positive effect of cue‐behavior repetition on habit strength, but not vice versa. The effect of habit strength on daily steps varied by the operationalization of cue‐behavior repetition. Tentative findings suggest that commitment and prompts and cues are effective habit‐promotion strategies when delivered together.

## INTRODUCTION

Regular physical activity has been consistently linked to a reduced risk of mortality from chronic diseases (Warburton & Bredin, 2017). Yet, nearly one‐third of adults worldwide fail to meet the recommended physical activity guidelines (Guthold et al., 2018). In this context, understanding the factors that influence physical activity and developing targeted strategies to enhance long‐term, sustained physical activity are critical steps toward improving population health.

It has been suggested that automatic processes play a critical role in sustaining health behaviors (Kwasnicka et al., 2016). These processes support behavioral maintenance by complementing self‐regulation, which relies on finite psychological resources that can be depleted (Hagger, 2019). Among automatic processes, *habit* has gained prominence for its positive association with physical activity (Feil et al., 2021; Gardner et al., 2011; Rebar et al., 2016) driving increased interest in physical activity promotion research (Hagger, 2019; Rhodes & Rebar, 2018).

### Habit, cue‐behavior repetition, and physical activity

Habit can be understood as a cognitive representation of a cue‐behavior association, acquired through repetition of the behavior in the presence of the cue (cue‐behavior repetition) (Fleetwood, 2021). Once established, a habit, in turn, promotes the likelihood of repeated execution of the behavior by automatically instigating the associated behavior when the cue occurs (Gardner, 2015). Thus, cue‐behavior repetition plays a key role in the habit formation process, serving as both an antecedent and an outcome of habit formation.

Previous studies have mostly examined how cue‐behavior repetition leads to habit formation, consistently finding a positive effect (Keller et al., 2021; Lally et al., 2010; van der Weiden et al., 2020). However, despite its theoretical and practical implications, the reverse association, with habit as an antecedent to cue‐behavior repetition, remains unexplored. Addressing this research gap is crucial as it allows for the investigation of the pathways through which habit formation leads to increased physical activity.

### Temporal trajectory of the habit formation process

Given the role of habit in promoting health‐related behaviors, previous research has explored the trajectory of habit formation. Existing findings suggest that habit formation typically follows a non‐linear pattern, often characterized by asymptotic growth, where initial rapid progress gradually slows and plateaus (Fournier et al., 2017; Gardner et al., 2022; Keller et al., 2021; Lally et al., 2010; van der Weiden et al., 2020). Other trajectories, such as the “inverted U‐shaped” quadratic model, have been used to indicate a discontinuation in the habit formation process (Keller et al., 2021). Aligning with recommendations for habit‐tracking research (Gardner et al., 2022), these studies examined the trajectory of habit formation by adopting a longitudinal design and accounting for individual differences by modeling person‐specific habit trajectories. These methodological aspects are critical for obtaining valid person‐specific descriptions of the habit formation process.

Despite these methodological strengths, we argue that previous research has not fully explored the various possibilities that might define the habit formation process. Specifically, the shape of the habit formation process (i.e., the trajectory) has typically been predefined by researchers through the selection of specific target functions (e.g., quadratic, asymptotic) (Keller et al., 2021; Lally et al., 2010). This approach constrains the trajectories of habit formation to a fixed set of non‐linear patterns, displaying no more than one or two bends, ultimately risking to overlook nuances and alternative patterns. Whether the habit formation process can be adequately described by such functions or is better represented by more flexible functions accounting for more complex and nuanced non‐linear patterns remains an open empirical question. Importantly, to model such non‐linear patterns, it is key to move towards more intensive assessments (Gardner et al., 2022) and ensure that the possibility of distinguishing between momentary fluctuations and valid trajectories is not affected by too many missing values or too few observations in the time series (e.g., Keller et al., 2021; van der Weiden et al., 2020).

### Intervention strategies to support habit formation

The existing literature emphasizes the value of developing interventions that promote behavior change through habit formation (Orbell & Verplanken, 2020). Therefore, it is important to identify effective strategies to promote habit formation. A recent meta‐regression of ten randomized controlled trials (RCTs) found that interventions promoting habit formation for physical activity have a small to medium effect size on habit strength (Cohen's *d* = 0.31) (Ma et al., 2023). Notably, the risk of bias across studies was low, but there was considerable heterogeneity (I^2^ = 64%). Given that habit forms after cue‐dependent repetition of the target behavior (Fleetwood, 2021; Gardner, 2015), habit interventions should help individuals identify the context and cues to be associated with the target behavior and facilitate the repetition of behavior at the occurrence of the cue (Gardner et al., 2020). This can be done by formulating implementation intentions (Gollwitzer, 1999). A key factor determining the effectiveness of implementation intentions, and thus the execution of the target behavior is their cognitive accessibility (Lally & Gardner, 2013; Tobias, 2009). Cognitive accessibility of an intended behavior depends on the salience of the cue itself but can also be supported by external memory aids. These aids increase the likelihood of remembering to perform the behavior during opportunities for action (Gardner & Rebar, 2019; Lally & Gardner, 2013; Tobias, 2009). Practically, this can be achieved by sending reminders (e.g., text messages, notifications) to reinforce implementation intentions, which have also been shown to have a positive effect on physical activity rates (e.g., brisk walking) (Prestwich et al., 2010).

Despite arguments in favor of using reminders to support habit formation, the meta‐regression by Ma et al. (2023) found no significant positive effect of the behavior change technique (BCT) *prompts and cues* (i.e., the broader category of environmental stimuli, including reminders, aiming at prompting or cueing the behavior; Michie et al., 2013) on habit formation in physical activity. These null findings might have theoretical and methodological explanations. Previous research has suggested that the effect of reminders decays over time (Tobias, 2009), meaning their impact might not be evident unless the interaction with time is taken into account. Additionally, the effect of reminders depends on the person's commitment to performing the target behavior (Tobias, 2009). Therefore, commitment, understood as the strength of the tension or decision towards performing an intended behavior (Inauen et al., 2014; Tobias, 2009), represents a prerequisite for the effectiveness of implementation intentions (Sheeran et al., 2005).

Another explanation for Ma and colleagues' null findings (2023) might be attributed to the inclusion criteria adopted in the meta‐regression. Specifically, RCTs are not designed to investigate which intervention components are efficacious, and this limitation is reflected in the absence of dosage considerations for the BCTs in the meta‐regression. This task is better suited for factorial trials, which are specifically designed to evaluate the individual effects of intervention components and to assess whether one component impacts the effect of another (Collins et al., 2014). An extension to factorial trials is represented by micro‐randomized trials (MRTs), an efficient design characterized by intensive longitudinal data and within‐person randomization of the intervention components (Klasnja et al., 2015). MRTs enable researchers to assess how the effects of intervention components change over time and how these components interact. Notably, MRTs would allow testing hypotheses such as whether the effect of reminders decays over time or whether reminders are more effective when combined with strategies aimed at increasing commitment.

### Aim of the study

In this study, we examined the temporal trajectory of habit formation in relation to physical activity and its relationship with cue‐behavior repetition (with the target behavior being a 15‐minute brisk walk) and unconditional physical activity (operationalized as daily step count) over time. We chose brisk walking as the target behavior due to its association with various health markers (Tudor‐Locke et al., 2018). We also aimed to investigate the effects of the BCTs prompts and cues, and commitment on habit strength, cue‐behavior repetition, and unconditional physical activity. The research questions, visually represented in the supplemental materials (Figure S1), were co‐registered^1^ at https://osf.io/cpbua.

#### RQ1 – habit strength as dependent variable

We examined the temporal trajectory of habit formation for physical activity, hypothesizing it to be best described by an asymptotic model (H1.1). Additionally, we investigated the effect of cue‐behavior repetition on habit strength, expecting a positive association (H1.2). We also examined the independent and joint effects of BCT prompts and cues and commitment on habit strength. Specifically, we hypothesized a positive main effect of prompts and cues (H1.3), commitment (H1.4), as well as their interaction effect (H1.5) on habit strength. Finally, we hypothesized that the effect of BCTs on habit strength decreases over time (H1.6).

#### RQ2 – cue‐behavior repetition as dependent variable

We investigated the effect of habit strength on cue‐behavior repetition, expecting a positive association (H2.1). Additionally, we examined the independent and joint effects of BCTs prompts and cues and commitment on cue‐behavior repetition. Specifically, we hypothesized a positive main effect of prompts and cues (H2.2), commitment (H2.3), as well as their interaction effect (H2.4) on cue‐behavior repetition.

#### RQ3 – unconditional physical activity as dependent variable

We examined the effect of BCTs prompts and cues and commitment on unconditional physical activity (daily step count) over time, expecting a positive association (H3.1). Finally, we hypothesized that this effect was mediated by habit strength and cue‐behavior repetition (H3.2).

## METHODS

This was a digital behavior change intervention delivered via the smartphone app HabitWalk. The study lasted 112 days and was organized into two phases: a preparatory phase (days 1–7) during which participants were supported in defining their implementation intentions, and an experimental phase (days 8–112) during which the BCTs prompts and cues, and commitment were experimentally manipulated. Specifically, this second phase was characterized by a micro‐randomized design (Klasnja et al., 2015). This involved daily within‐person randomization of each BCT, each with a 50% probability of being presented to the participants on any given day. The study took place between May 5th and October 31st, 2023, in Switzerland.

### Participants

The target population consisted of inactive (i.e., performing less than 150 min of moderate or 75 min of vigorous physical activity per week) adults (age ≥ 18) from the general Swiss population who intended to increase their daily steps during a 15‐week digital intervention. Persons who did not own an Android smartphone or an iPhone with Internet access were not willing to uninstall the Garmin connect app, were pregnant or had pre‐existing health conditions (e.g., cardiovascular disease), or did not give informed consent were excluded.

The target sample size for this study was set at 34 participants, each ideally generating 105 observations. Due to its intensive longitudinal nature and the MRT design, statistical power does not only rely on the number of participants but also on the number of observations per participant (Klasnja et al., 2015). We defined this sample size as it allows to test the current hypothesis using both an n‐of‐1 (idiographic) and a multilevel approach. For the n‐of‐1 approach, where each participant is considered an analytical unit, a time series of at least 70 observations is required for accurate estimation and inference, with 100 observations representing the optimal parsimonious length (Stadnitski, 2020). For the multilevel approach, a sample of 34 participants × 105 observations, was enough to detect a small to medium effect size. This power analysis was calculated using the R *simr* package (Green & MacLeod, 2016) with the code adapted from Arend and Schäfer (2019). Finally, anticipating a dropout rate of around 15%, we planned to recruit 40 participants. However, due to some participants dropping out during the preparatory phase, we continued recruitment until 40 participants entered the experimental phase.

### Measures

#### Baseline measures

The following variables were measured with a baseline questionnaire: age, height, weight, sex, and sociodemographic details (e.g., employment, living situation; see Table S1 in the supplemental materials). Baseline physical activity was assessed with the International Physical Activity Questionnaire (IPAQ‐SF) (Craig et al., 2003).

#### Time series measures

##### Habit strength

During the experimental phase, habit strength was measured daily in the evening using the German version of the Self‐Report Behavioral Automaticity Index (SRBAI) (Gardner et al., 2012; Verplanken, 2007). The SRBAI consists of four items from the Self‐Report Habit Index (Verplanken & Orbell, 2003). The SRBAI was phrased to reference the cue selected by the participant (in brackets) “*Walking for 15 minutes after my cue [chosen cue] is something that …*” followed by four statements (a) “*I do automatically*”, (b) *“I do without having to consciously remember”*, (c) *“I do without thinking”*, and (d) “*I start doing before I realize I'm doing it*”. The response scale ranged from ‘does not apply at all’ (1) to ‘applies completely’ (5). The SRBAI displayed high reliability for between‐person averages (R_KF_ ≈ 1; Cranford et al. 2006) and to detect within‐person change (R_C_ = 0.82; Scott et al. 2020).

##### Cue‐behavior repetition

Cue‐behavior repetition reflects the repetition of a target behavior in response to a specific cue. We defined the target behavior as a 15‐minute brisk walk session prompted by the cue that each participant chose. Participants were instructed to initiate recording an activity session on their Garmin Vivosmart 4 activity tracker when they began the brisk walk after encountering the cue. Vivosmart 4 is a wrist‐worn device from Garmin (Garmin Ltd., Olathe, Kansas) with high validity in measuring step count (Evenson & Spade, 2020). During the activity session, the Vivosmart 4 provides real‐time information, such as duration and steps taken. Cue‐behavior repetition was operationalized as any user‐registered activity lasting at least one minute, leading to a binary variable (0 = no recorded activity; 1 = recorded activity). This operationalization does not differentiate instances of ‘no recorded activity’ based on whether the participants wore the device or not on a given day. Recorded activity sessions provided metrics such as duration, start and end times, and steps taken. For sensitivity analysis, we tested our hypotheses under different operational definitions of cue‐behavior repetition based on some key features of the target behavior (i.e., duration and cadence of the walk, proximity of the walk to the expected cue time) (see Box S1 in supplemental materials).

##### Unconditional physical activity

Unconditional physical activity was operationalized as daily step count, continuously assessed using the Garmin Vivosmart 4 activity tracker. Cumulative step counts for the day were accessible to participants at any time by swiping the screen of the device.

### Experimental manipulation

During the experimental phase, the BCTs prompts and cues and commitment were delivered to the participants following a daily within‐person randomization at the time specified by the participants at the end of the preparatory phase. Each BCT had a 50% chance of being presented to participants each day, leading to days where they received none, one, or both BCTs (see supplemental materials, Figure S2). Randomization was performed by the HabitWalk app and blinding was ensured. The BCT prompts and cues was implemented as a reminder in the form of a push notification sent by the HabitWalk app to the participant at the time they set during the preparatory phase. The push notification contained the following message: *“Soon it will be time for your 15‐minute brisk walk after [your cue]. Get ready!”*. The BCT commitment was implemented as a morning‐guided task. First, participants were advised to write a short sentence committing to their goal and motivating themselves to follow their implementation intentions. Second, they were asked to rate, on a 5‐point Likert scale, how personally important they rated taking a 15‐minute brisk walk after their chosen cue (1 – not important, 5 – very important), and their sense of obligation to themselves regarding this commitment (1 – not at all, 5 – completely). These two items aimed to enhance commitment rather than measure it (i.e., question‐behavior effect; Wilding et al., 2019).

### Procedure

The study was conducted through the HabitWalk app, which was developed for this study. The app delivered the interventions and assessed all outcome measures. Specifically, the app measured habit strength through a daily diary and retrieved physical activity data (i.e., brisk walking sessions and daily steps) collected by two other technological components: the Garmin Vivosmart4 activity tracker and the Fitrockr app (https://www.fitrockr.com/) (see Box S2 in supplemental materials).

Participants were recruited via social media platforms and leaflets from May to July 2023. Interested individuals filled out a screening questionnaire, and those eligible were invited for an on‐site appointment. During this appointment, they provided informed consent and installed the technological infrastructure. After the on‐site appointment, participants filled out the baseline survey via the HabitWalk app and entered the preparatory phase (day 1). Right after completing this survey, participants were introduced to the concepts of habit, habitual behavior, and implementation intentions. Then, they were instructed by the app to observe themselves over the following few days, identifying cues suitable for initiating a daily 15‐minute brisk walk. Participants were told that cues were suitable if they: *i*) occurred once daily (e.g., having lunch), *ii*) allowed for the walk to occur, *iii*) corresponded to an approximate time, and *iv*) were not already linked to a habitual physical activity routine. This latter point was specified to ensure that participants did not already have a physical activity habit associated with the identified cues. Additionally, participants were provided with examples of routine‐based cues. From day 2 to 5, participants were prompted to note down potential candidate cues in the app and were also encouraged to assess the feasibility of performing a 15‐minute brisk walk at the occurrence of these cues. This preparatory task lasted for a few days as the cue selection process deserves particular attention due to its subjective meaning and inherent complexity (Rebar et al., 2024).

During days 6 and 7, participants were asked to choose a final cue for their implementation intentions to be used during the next 15 weeks (i.e., the experimental phase). They were guided to create an if‐then plan to link the initiation of the 15‐minute brisk walk to the chosen cue, for example, “*When I finish lunch, then I will go out for a 15‐minutes brisk walk”*. For the reminder, they were asked to specify their preferred reminder time, aiming to closely align it with the expected time of their cues to ensure timely delivery (i.e., a maximum of half an hour before the cue). They were also asked to enter the time at which they usually wake up, allowing the app to deliver the commitment task to be completed in the morning before the cue occurs. Additionally, participants selected a time range (5 p.m. to 10:30 p.m.) for receiving reminders to fill out the daily SRBAI questionnaire. The day after completing the preparatory phase, participants entered the experimental phase. During this 15‐week phase, participants were asked to record their daily 15‐minute brisk walks when triggered by the chosen cue and stop recording when they finished walking. During this phase, the BCTs were delivered as per experimental manipulation.

At the end of the study, participants were invited to return the activity tracker and received a debriefing. Participants were reimbursed up to a maximum of 100 CHF for their participation, with the final amount depending on the number of daily diaries they filled out.

### Data analysis

Analyses were conducted with the R software, version 4.3.2 (R Core Team, 2021). R code and data are available at: https://osf.io/58pbr/.

#### Data preparation

On some days, participants recorded cue‐behavior repetitions more than once, which contradicts our instructions to identify a cue that occurs once daily. In such instances, we kept the recording that occurred closest to the chosen cue time. For daily step data, as in previous research (Chevance et al., 2021), days with step counts below 500 were defined as non‐wear days and recoded as missing values, as they likely represent days when the activity tracker was only partially worn. Second, person‐specific outlying observations were winsorized by replacing values below the 5th percentile with the value at the 5th percentile and values above the 95th percentile with the value at the 95th percentile (Aguinis et al., 2013).

#### Time series characteristics and missing values

Participants entering the intervention phase had an average of 50.1 missing days (range = 2–105 days) in habit strength, 60.8 missing days (range = 5–105 days) in cue‐behavior repetition, and 43.9 missing days (range = 4–105 days) in daily steps (see supplemental materials, Table S2). To preserve both internal and external validity, time series with over 50% missing values in habit strength during the initial 66 days of the intervention were excluded from the analysis to ensure data quality and enable a detailed examination of the habit strength trajectory. This threshold was chosen based on previous research suggesting that approximately 66 days are needed for habit to form (Keller et al., 2021; Lally et al., 2010). Missing values in habit strength and daily steps were imputed with the *imputeTS* package (Moritz & Bartz‐Beielstein, 2017) using the Kalman Filter method, recommended for univariate time series imputation (Gómez & Maravall, 1994), only if the missing value was preceded by an actual observation (see supplemental materials, Figure S3). Attempts to impute longer missing gaps resulted in an unrealistic decrease in imputed value variance. No imputation was applied to cue‐behavior repetition, as a missing observation indicated that cue‐behavior repetition did not occur.

#### Main analysis

The temporal trajectory of habit strength (H1a) was modeled adopting an idiographic approach (i.e., each participant separately) to account for individual differences in the growth trajectory (Gardner et al., 2022). For each participant, we modeled the trajectory with five models: constant, linear, quadratic, asymptotic, and generalized additive (GAMs) (for further details on growth models' specification, refer to supplemental materials, Table S3). The first four models come from previous habit‐tracking studies (Edgren et al., 2024; Fournier et al., 2017; Keller et al., 2021; Lally et al., 2010). GAMs were introduced as they allow fitting smoothed functions by combining multiple low‐level functions, ultimately providing a flexible approach to model non‐linear trends that account for multiple and complex bends. GAMs were run with the *mgcv* package (Wood, 2017). The best model selection for each participant was based on the Bayesian Information Criterion (BIC) with lower values indicating better performance (Chakrabarti & Ghosh, 2011).

We utilized multilevel models (MLM) and implemented them using the *lme4* package (Bates et al., 2015) to test the remaining hypotheses. MLM is an appropriate method for handling nested data structures, such as daily habit strength observations within participants. This approach enables the estimation of average (fixed) and person‐specific (random) effects. Specifically, we examined the fixed effects of: *i*) cue‐behavior repetition, BCTs prompts and cues, and commitment (independent variables; IVs) on habit strength as a dependent variable (DV) (H1.2 – H1.7) with MLM 1; *ii*) habit strength, BCTs prompts and cues and commitment (IVs) on cue‐behavior repetition as DV (H2.1 – H2.4) with MLM 2; and *iii*) habit strength, cue‐behavior repetition, and BCTs prompts and cues and commitment (IVs) on daily steps as DV (H3.1 – H3.1) with MLM 3. Continuous predictors, with the exception of time, were person‐mean centered to disaggregate between‐ and within‐person effects (Curran & Bauer, 2011). The effects of the BCTs prompts and cues and commitment were modeled using the ‘Intention to Treat’ approach, which means they were operationalized based on whether they were randomized on a given day. Additionally, we considered modeling seasonal effects for daily steps in MLM 3, but refrained from doing so as adding seasonal variables (i.e., dummy month variables) did not improve the model.

The inclusion of random effects was based on iterative comparisons of the BIC values between pairs of nested models, with lower BIC values indicating better model performance. For MLM 1, a random intercept (participant) and random slopes (time, cue‐behavior repetition, autoregressive parameter) were included. For MLM 2, both a random intercept (participant) and random slope (time) were included. For MLM 3, only the random intercept (participant) was included. See Table S4 in the supplemental materials for the detailed specification of the models.

For sensitivity analysis, MLM 1 was also run without cue‐behavior repetition as a predictor to eliminate it as a plausible mediator of the BCTs' effects on habit strength. Similarly, MLM 3 was also run without both cue‐behavior repetition and habit strength as predictors to eliminate them as plausible mediators of the BCTs' effects on daily steps. For details on divergence from registered analysis see supplementary materials (Box S3).

## RESULTS

### Preliminary analysis

A total of 47 participants were recruited. Among them, 2 (4.26%) did not start the preparatory phase, and 5 (10.64%) did not complete it, resulting in 40 participants defining their implementation intentions and entering the experimental phase (the list of selected cues for the implementation intentions is included in the supplemental materials, Tables S5–S6). Of these, 16 participants had more than 50% missing values in the habit strength time series during the initial 66 days of the intervention and were therefore excluded from the current analysis. This led to a final analytical sample of 24 participants. Notably, 12 participants out of the 40 who entered the experimental phase requested to stop their study participation (see Table S7). Of these, three were included in the analytical sample because their data met the inclusion criteria regarding missing value frequency. The participant flow is available in the supplemental material (Figure S4).

The sociodemographic characteristics of all the recruited participants who filled out the baseline survey (*N* = 45) and those included in the final analytical sample (*n* = 24) are displayed in Table S1 in the supplemental materials. Among the analytical sample, the median age of the participants was 41 years, and 22 (92%) were women. Most of them lived with their family (*n* = 10, 42%) or partner (*n* = 8, 33%). The majority had completed vocational training or university studies (*n* = 21, 88%), and was either employed (*n* = 18, 75%) or studying (*n* = 4, 17%). The median Body Mass Index (BMI) of the participants was 23.9. In terms of physical activity, participants in the analytical sample had a median of 1,497 MET minutes per week, and 67% of them could be classified as moderately active (see study limitations).

A dropout analysis was conducted to examine baseline differences between the participants in the analytical sample (*n* = 24) and those excluded from the analysis (*n* = 21) (see Table S1 in the supplemental materials). The results suggest that the two groups did not differ on any of the variables measured at baseline, with the exception of employment after aggregating the variable into two categories (‘employed’ vs. ‘others’). The results indicate that included participants were significantly more likely to be employed than dropouts (*p* = .037). Finally, although not significant, it is worth mentioning that the analytical sample had about twice as many MET‐minutes/week (median = 570) of moderate‐intensity physical activity compared to the dropouts (median = 240).

The analysis of fidelity of treatment receipt indicated that participants completed the commitment task 51.5% (median) (range = 0–91%) out of a total of 53 days during which the BCT commitment was randomized and delivered to each person. If we consider only those days during which participants filled out the daily diary with the SRBAI assessment, the median percentage of completed tasks increased to 60.5% (range = 0–92%). The information on the ratio between delivery and usage is not available for the BCT prompts and cues, as we could not verify whether and when participants checked the push notifications.

### Main analysis

#### The shape of the temporal trajectory of habit formation

**FIGURE 1:**
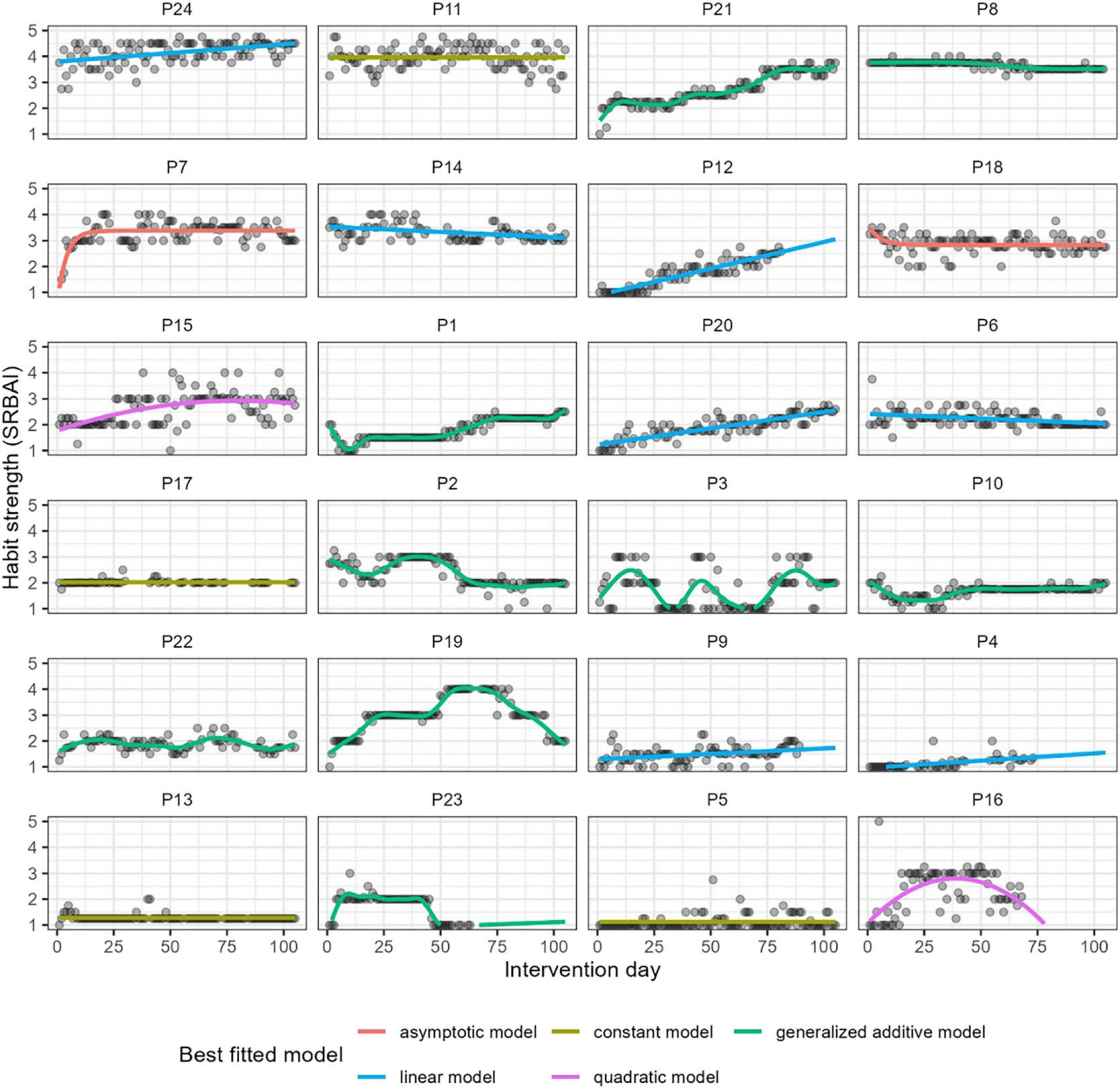
Person specific trajectories in habit strength. *Note*: Each panel plot displays the best‐fitting habit trajectory for one participant (P). Time series are ordered by the last predicted value in habit strength (SRBAI), from highest to lowest.

Out of the 24 habit strength time series, model comparison for each participant revealed that the trajectory of habit strength was best described by a constant model in four cases (17%), a linear model in seven cases (29%), a quadratic model in two cases (8%), an asymptotic model in two cases (8%), and more complex models (GAMs) in nine cases (38%) (see Figure 1). Seven participants (29%) had the last predicted value of the SRBAI above the scale midpoint (see supplemental materials, Table S8).

#### Effect of cue‐behavior repetition, prompts and cues and commitment on habit strength

The intraclass correlation coefficient (ICC = 0.9) indicated that a high proportion of the variance in habit strength (90%) was explained by stable between‐person differences (see MLM 1, Table 1). Multilevel analysis suggested a positive effect of cue‐behavior repetition on habit strength (*B* = 0.16, 95% CI = [0.08, 0.23], β = 0.08), confirming our hypothesis (H1.2). Contrary to our hypotheses, we did not find any effects of the BCT prompts and cues (H1.3) or commitment (H1.4) on habit strength. However, their joint effect, measured via their interaction term, was positively associated with habit strength, confirming H1.5. This significant association disappeared when cue‐behavior repetition was removed from the model predictors. This post‐hoc deviation from the study protocol aimed to eliminate a plausible mediator of the effects of the BCTs on habit. Additionally, contrary to our hypothesis (H1.6), the effect of the BCTs on habit strength did not decrease over time. Lastly, we detected a significant and positive autoregressive parameter at lag‐1 (*B* = 0.35, 95% CI = [0.23, 0.46], β = 0.16), showing an association of habit strength of the previous day on the current day.

**TABLE 1:** Multilevel models summary.

	Registered models			Models without plausible mediators		
	Fixed effect		Random effect	Fixed effect		Random effect
Model 1 – DV is habit strength	Est. (SE)	95% CI	SD	Est. (SE)	95% CI	SD
**Intercept**	**2.16 (0.19)**	**[1.79, 2.54]**	0.91	**2.28 (0.19)**	**[1.90, 2.66]**	0.92
**Time**	**<0.01 (0.00)**	**[0.00, 0.00]**	<0.01	**<0.01 (0.00)**	**[0.00, 0.00]**	<0.01
**HS**_**t‐1**_	**0.35 (0.06)**	**[0.23, 0.46]**	0.25	**0.36 (0.06)**	**[0.24, 0.48]**	0.26
**CBR**_**t**_	**0.16 (0.04)**	**[0.08, 0.23]**	0.17	‐	‐	‐
P&C_t_	−0.05 (0.03)	[−0.11, 0.00]	‐	−0.05 (0.03)	[−0.11, 0.01]	‐
Commitment_t_	−0.03 (0.03)	[−0.09, 0.03]	‐	−0.04 (0.03)	[−0.10, 0.03]	‐
**P&C**_t_ **:Commitment**_t_	**0.06 (0.03)**	**[0.00, 0.11]**	‐	0.05 (0.03)	[−0.00, 0.11]	‐
Time:P&C_t_	<0.01 (0.00)	[−0.00, 0.00]	‐	<0.01 (0.00)	[−0.00, 0.00]	‐
Time:Commitment_t_	<0.01 (0.00)	[−0.00, 0.00]	‐	<0.01 (0.00)	[−0.00, 0.00]	‐
Number observations = 2,107; ICC = 0.9							
**Model 2 – DV is CBR**	**OR (SE)**	**95% CI**	**SD**	**OR (SE)**	**95% CI**	**SD**
**Intercept**	**2.64 (0.78)**	**[1.49, 4.86]**	3.22	‐	‐	‐
**Time**	**0.99 (0.00)**	**[0.98, 0.99]**	1.01	‐	‐	‐
**CBR**_**t‐1**_	**1.52 (0.18)**	**[1.20, 1.92]**	‐	‐	‐	‐
HS_t‐1_	1.13 (0.14)	[0.88, 1.45]	‐	‐	‐	‐
P&C_t_	1.08 (0.16)	[0.81, 1.45]	‐	‐	‐	‐
Commitment_t_	1.11 (0.16)	[0.83, 1.48]	‐	‐	‐	‐
P&C_t_:Commitment_t_	1.07 (0.22)	[0.61, 1.65]	‐	‐	‐	‐
Number observations = 2,184; ICC = 0.3							
**Model 3 – DV is steps**	**Est. (SE)**	**95% CI**	**SD**	**Est. (SE)**	**95% CI**	**SD**
**Intercept**	**6289.99** **(520.02)**	**[5260.26, 7310.52]**	2089.45	**7830.53 (485.70)**	**[6862.66, 8785.71]**	2045.73
Time	2.91 (3.27)	[−3.49, 9.29]	‐	0.78 (3.20)	[−5.49, 7.03]	‐
**Steps**_**t‐1**_	**0.18 (0.02)**	**[0.14, 0.23]**	‐	**0.17 (0.02)**	**[0.13, 0.22]**	‐
HS_t_	330.78 (201.81)	[−64.40, 725.51]	‐	‐	‐	‐
**CBR**_**t**_	**2123.09 (220.18)**	**[1691.61, 2553.39]**	‐	‐	‐	‐
P&C_t_	−115.30 (248.98)	[−602.73, 371.83]	‐	25.84 (251.10)	[−465.97, 517.51]	‐
Commitment_t_	−43.56 (246.28)	[−525.89, 438.13]	‐	117.18 (249.89)	[−372.43, 606.32]	‐
P&C_t_:Commitment_t_	63.84 (349.91)	[−620.46, 749.18]	‐	8.61 (354.59)	[−685.53, 703.28]	‐
Number observations = 1742; ICC = 0.2						

*Note*: Significant fixed effects are in bold; OR = odds ratio from GLMER models; DV = dependent variable; HS = habit strength; CBR = Cue‐Behavior Repetition; P&C = Prompts and Cues.

#### Effect of habit strength, prompts and cues and commitment on cue‐behavior repetition

The ICC of 0.3 indicated that 30% of the variance in cue‐behavior repetition was explained by stable between‐person differences (see MLM 2, Table 1). Results from the multilevel analysis rejected all our hypotheses. Notably, we did not find any positive effect of habit strength on cue‐behavior repetition (H2.1) nor the independent or interaction effects of BCT prompts and cues and commitment on cue‐behavior repetition (H2.2 – H2.4). Again, we found a significant and positive autoregressive parameter at lag‐1 (*OR* = 1.52, 95% CI = [1.20, 1.92], β = 1.22), suggesting that the repetition of the target behavior was more likely to occur if it also occurred on the previous day.

#### Effect of habit strength, cue‐behavior repetition, prompts and cues, and commitment on steps

The ICC of 0.2 indicated that only 20% of the variance in steps was explained by between‐person differences (see MLM 3, Table 1). Results did not confirm the expected positive effect of BCT prompts and cues and commitment on daily step count (H3.1) (see model 3 results without the inclusion of cue‐behavior repetition and habit strength as model predictors), thus also rejecting the hypothesis that their effect is mediated by habit strength and cue‐behavior repetition (H3.2). When examining the association between habit strength, cue‐behavior repetition, and daily steps, multilevel analysis suggested no effect of habit strength on daily steps but a positive effect of cue‐behavior repetition on daily steps (*B* = 2123.09, 95% CI = [1691.61, 2553.39], β = 0.22). Finally, we also found a positive autoregressive parameter at lag‐1, suggesting that the number of steps on one day had a meaningful influence on daily steps the following day.

### Sensitivity analysis

We conducted various sensitivity analyses to corroborate the robustness of the current findings but also to assess the impact of different operational choices on the testing of the study hypotheses. In the first set of sensitivity analyses, we tested our hypotheses under different operational definitions of cue‐behavior repetition. Results provided in the supplemental materials (Tables S9–S11) generally confirmed the current findings from MLM 1 and 2. For MLM 3, we found a positive association between habit strength and daily steps under all other operationalizations of cue‐behavior repetition. In a second set, we coded cue‐behavior repetition as ‘missing’ instead of 0 for entries where the activity was not recorded due to the device not being worn. The results confirmed most of the findings from the main analysis, except for the interaction effect of prompts and cues and commitment on habit strength. Although the effect size remained the same, it was no longer statistically significant (see Table S12).

In a third set, we operationalized cue‐behavior repetition by combining the Garmin‐recorded activity with participants' daily evening reports. The results confirmed most of the findings from the main analysis and also revealed a positive effect of prompts and cues on cue‐behavior repetition. Additionally, they corroborated the first set of sensitivity analyses, showing a positive effect of habit strength on daily steps (see Table S13).

Fourth, we re‐ran MLM 1–3 with all participants who entered the intervention phase. The results confirmed the main analysis with the only difference being that we also found a negative effect of prompts and cues on habit (see Table S14).

Finally, we provided n‐of‐1 level results for participants (*n* = 16) with less than 20% missing values in the habit strength time series. The most relevant findings indicated that for 9 out of 16 participants, cue‐behavior repetition was a significant predictor of habit strength. However, it should be noted that some participants exhibited very high occurrences of cue‐behavior repetition, resulting in limited variance in this variable. Another notable finding concerns the interaction effects of the BCTs on habit strength, which was significant only for a few participants (see Tables S15–S17 and Figures S5–S7 for a more detailed overview of the n‐of‐1 results).

## DISCUSSION

This study examined the temporal trajectory of habit formation in relation to physical activity and its relationship with cue‐behavior repetition and unconditional physical activity. We also tested whether prompts and cues and commitment could promote habit formation. Our findings confirmed that habit forms in a highly idiosyncratic manner. Extending previous findings, the formation process was only seldom characterized by asymptotic growth. We observed a positive effect of cue‐behavior repetition on habit strength, but not vice versa. Additionally, we found that the effect of habit strength on unconditional physical activity varied depending on the operationalization of cue‐behavior repetition. Finally, tentative findings suggest that commitment and prompts and cues are effective BCTs to promote habit strength within persons, but only when delivered together.

### How does habit strength change over time?

In line with a previous review (Gardner et al., 2022), we found the trajectory of habit strength changes over time in a highly idiosyncratic and often non‐linear manner. Notably, in 17 out of 24 participants (71%), the last predicted habit value was below the scale midpoint, indicating that by the end of the intervention, the habit remained weak (e.g., participant P20) or had increased and then declined (e.g., P19). Only 29% of participants (*n* = 7) had habit values above the scale midpoint by the end of the intervention. Among those, only one case (P7) confirmed the hypothesis that habit grows asymptotically. In other cases, change in habit was described by constant (P11), linear (P24, P12), or more complex (P21, P8) trajectories. Additionally, consistent with previous research (Keller et al., 2021; van der Weiden et al., 2020), the very high ICC was mostly characterized by stable between‐person differences, which underscores that habit is hard to change. One consideration for future research based on the high ICC is to include group‐level predictors in the model to better understand how group characteristics impact habit.

There are some differences from previous habit‐tracking studies regarding the shape of the habit strength trajectory, which can be attributed to methodological aspects. First, unlike previous research (Keller et al., 2021; Lally et al., 2010; van der Weiden et al., 2020), our study's target behavior was not self‐selected by participants but was defined by design. Therefore, even though participants were aware of the target behavior from the moment they completed the screening questionnaire, it might have been difficult to incorporate it into their routine. This is supported by the fact that for some participants, the increase in habit strength was still in place at the end of the study (e.g., P20, P21). Future research should address this and examine the implication of self‐selected vs. assigned target behaviors on the habit formation trajectory. Second, in our study, we found a few instances where the fitted values at the beginning of the experimental phase were already high. High baseline scores in habit strength might be because these participants identified the cue already at the very beginning of the preparatory phase and started developing the habit during that phase when they were prompted to find and experiment with the cue to trigger the brisk walk and assess its feasibility (see Table S5 in the supplemental materials). Therefore, we might not have been able to model the initial rapid increase that characterizes the hypothesized asymptotic curve. Finally, by including GAMs in our modeling approach, we empirically tested that the process of change in habit strength can sometimes be better described by models allowing for multiple or different types of bends that otherwise would have been overlooked or labeled differently.

Since various alternative functions are needed to account for heterogeneity in habit strength trajectories (i.e., the functions used in the current study, but see Edgren et al., 2024, for additional functions worth exploring, such as the logistic one), future research should go beyond identifying the best‐fitting functions and aim to harmonize the different trajectories to increase their practical utility. For example, researchers may extract the first‐order derivative of the various growth functions, which provides key information (i.e., rate of change) on the progress throughout the habit formation process, ultimately indicating whether habit strength is increasing, decreasing, or remaining stable at each time point. Working in this direction, or leveraging other less conventional methodological approaches in habit research (e.g., computational models; Rebar et al., 2024; Tobias, 2009), would also allow modeling how the habit trajectory changes in relation to other key variables such as cue‐behavior repetition.

### What is the reciprocal effect between Cue‐behavior repetition and habit strength, and how do they relate to daily steps?

Our results confirmed previous literature (Keller et al., 2021; Lally et al., 2010; van der Weiden et al., 2020), indicating a positive effect of cue‐behavior repetition on habit strength, which is also confirmed in all our sensitivity analyses and in 9 out of 16 participants included in the n‐of‐1 analysis. However, compared to Keller et al. (2021) and van der Weiden et al. (2020), we detected a smaller effect size. A plausible explanation for this difference may be attributed to several contextual and methodological factors that are worth mentioning, as they contribute to defining the boundary conditions for estimating the current effect of cue‐behavior repetition on habit strength. First, behavior specificity might have played a role: unlike previous studies focusing on various health‐related behaviors (Keller et al., 2021; Lally et al., 2010; van der Weiden et al., 2020), we specifically examined the habit in relation to brisk walking, which may be considered a more complex target behavior compared to those used in prior research. Second, in contrast to Keller et al. (2021), we included the habit value from the previous day as model predictor, which accounted for a significant portion of the variance. Third, in our study, participants were invited to choose a cue that occurs no more than once per day, leading to a maximum of one daily repetition of the target behavior. More frequent repetitions of the target behavior per day (e.g., van der Weiden et al., 2020) might lead to a stronger effect. Last, our study differs from previous research (Keller et al., 2021; Lally et al., 2010; van der Weiden et al., 2020) in that we did not measure cue‐behavior repetition using a self‐report approach but opted for a more objective measure to limit biases related to self‐report measures (Althubaiti, 2016).

Contrary to our hypothesis, we did not find a positive lagged effect of habit strength on cue‐behavior repetition (see also the sensitivity analysis in Tables S12–S14, S16), meaning the association between conditional physical activity and habit strength is not reciprocal. Additionally, with the co‐registered operationalization of cue‐behavior repetition, we did not find the effect of habit on daily steps (i.e., unconditional physical activity), which was also confirmed by the n‐of‐1 analysis which adopted the same operationalization. However, sensitivity analysis indicated a positive effect of habit on daily steps across all other operationalizations of cue‐behavior repetition (Table S11). With necessary caution, these findings seem to suggest that although habit did not influence cue‐behavior repetition, it might still lead to increased daily steps. These results underscore the need for future studies to examine and disentangle the effects of habit strength on cue‐behavior repetition and unconditional physical activity. If the effect of habit on cue‐behavior repetition remains unsupported while its effect on unconditional physical activity persists, it raises further research questions in habit and physical activity research, particularly regarding the mechanisms through which habit enhances physical activity. A plausible explanation could be attributed to individuals' action control (Sniehotta, 2009): individuals monitored their behavior and, if the target brisk walk was not triggered by the cue, they may have compensated by incorporating cue‐independent walks at other times of the day.

### What is the effect of the BCTs prompts and cues on habit strength, Cue‐behavior repetition, and daily steps?

We did not find the main effects of commitment and prompts and cues individually on habit, confirming previous research (Ma et al., 2023; but see sensitivity analysis in Table S14 showing a negative effect of prompts and cues on habit strength when we analyzed all the 40 participants who entered the intervention phase). However, we found a combined effect of these BCTs in line with research suggesting that the effects of prompts and cues (reminders) depend on commitment to goal intentions (Gollwitzer, 1999; Tobias, 2009). A plausible theoretical explanation for this result is that intervening on the subjective importance of the intended behavior leads to cognitive tension, which facilitates memory processes (Tobias, 2009). Nonetheless, even though these results are confirmed in two out of three sensitivity analyses (Tables S12–S14), they should be considered tentative, as the interaction effect of the BCTs on habit strength was no longer significant after removing cue‐behavior repetition from the model predictors. Additionally, results from n‐of‐1 models (Table S15, Figure S5) seem to suggest that this interaction effect is idiosyncratic and its significance is mostly driven by a few participants with strong associations (P3, P11, P18). Further, it is important to note that the BCTs were not tested as standalone interventions, but were combined with implementation intentions, which served as the foundational intervention strategy and might have created a ceiling effect. A further point to consider when interpreting the current null findings is the participants' responsiveness to the BCTs. The commitment task was completed a median of 60.5% of the time when participants also reported their habit strength. This component, along with the reduced sample size due to attrition, might have increased the risk of false negatives. Results did not confirm the hypothesis of a positive effect of the BCTs on cue‐behavior repetition (H2.2 – H2.4; but see sensitivity analysis Table S13 showing a positive effect of prompts and cues on cue‐behavior repetition). Similarly, we did not find a positive effect of the BCTs on physical activity, thereby not establishing the prerequisite for the mediating role of habit and cue‐behavior repetition. In this regard, our findings do not corroborate previous literature (Prestwich et al., 2010).

### Strengths and limitations

A major strength of this study is its adherence to recent methodological recommendations for investigating the process of habit formation in ecological settings (Gardner et al., 2022). Additionally, to the best of our knowledge, this is the first study to measure cue‐behavior repetition without relying on retrospective self‐report measures, which are susceptible to biases (Althubaiti, 2016). Instead, we used a more objective and real‐time approach with participants recording the target brisk walking behavior with the activity tracker. This method also enabled us to conduct a series of rigorous sensitivity analyses, accounting for various features of cue‐behavior repetition (such as cadence, completion of the brisk walk, and proximity to the expected cue time) that would have been challenging, if not impossible, to assess via self‐report measures. Testing our hypotheses under different operationalizations of cue‐behavior repetition helped define the boundary conditions for the phenomena underlying the habit formation process. Finally, an important strength of this study is the micro‐randomized design, which allowed for testing the individual and combined effects of the BCTs, as well as whether these effects changed over time within persons.

This study is subject to certain limitations. First, we encountered a higher‐than‐expected rate of missing values in the habit strength time series, which ultimately resulted in an analytical sample smaller than originally planned. We addressed this challenge by prioritizing multilevel models instead of n‐of‐1 analyses, as they handle missing values better. Nonetheless, the presence of missing values constrained our ability to detect small to medium effect sizes accurately. Dropout analysis revealed no significant differences between the analytical sample and those excluded from the analysis, except for employment status. Additionally, despite the non‐significance of the test, the analytical sample exhibited levels of moderate physical activity more than twice as high as those of the dropouts and outside the boundaries of our inclusion criteria. This issue arose because the inclusion criterion of being physically inactive was operationalized as a single item in the screening questionnaire, and for 31 participants (69%), this answer was not confirmed by the results of the IPAQ questionnaire assessed a few days later during the baseline questionnaire. Therefore, current findings are representative of a population characterized by a higher physical activity profile and cannot be generalized to inactive adults. Another limitation is the use of a 500‐step threshold to identify non‐wear days, as employed in previous literature (e.g., Chevance et al., 2021). This method assumes low step counts reflect partial device wear. Heart rate data would have provided more accurate identification of non‐wear days but was not available.

## CONCLUSIONS

The study extends previous research on habit formation in physical activity. It confirms that habit formation is highly idiographic and highlights the critical role of cue‐behavior repetition in this process. In line with theory, tentative findings suggest that combining reminders with commitment tasks might be an effective strategy to support habit, although further research is needed to confirm this. Additionally, it introduced new approaches to operationalizing and measuring cue‐behavior repetition in habit research and systematically tested their impact on the observability of phenomena of interest, such as their relationships with habit and physical activity. Finally, this study identified areas for future research, particularly investigating the impact of self‐selected versus assigned target behaviors and elucidating the pathways through which habit influences increased physical activity.

## AUTHORS CONTRIBUTIONS

DB: Conceptualization, Funding acquisition, Data curation, Project administration, Resources, Formal analysis, Methodology, Visualization, Writing – original draft, Writing – review & editing. NG: Data curation, Project administration, Formal analysis, Visualization, Writing – original draft, Writing – review & editing. RE: Methodology, Writing – review & editing. JI: Conceptualization, Methodology, Writing – review & editing.

## FUNDING STATEMENT

This study was funded by the Suzanne und Hans Biäsch Foundation (u/Ref: 2022‐02).

## ETHICS APPROVAL STATEMENT

The Ethics Committee of the Faculty of Human Sciences at the University of Bern granted ethical approval for the study (Nr. 2023‐02‐05).

## CONFLICT OF INTEREST STATEMENT

The authors have no conflicts of interest to declare.

## Supporting information


**Data S1.** Supporting Information.

## Data Availability

Core R‐scripts and data are available online at https://osf.io/58pbr/.

## ENDNOTE

1 While the term “pre‐registration” describes a registration that occurs before data collection, “co‐registration” refers to a registration that happens contemporaneously with data collection but before viewing or analyzing the data (Benning et al., 2019). In this study, less than 10% of the data had been collected when the study was co‐registered.
