# Supplementary material for: HabitWalk: A micro‐randomized trial to understand and promote habit formation in physical activity
Source: Appl Psychol Health Well Being. 2024 Oct 10;17(1):e12605. doi: 10.1111/aphw.12605 (PMC11635918; doi:10.1111/aphw.12605)
Supplement: Supplementary file 1 — Data S1. Supporting Information. [file APHW-17-0-s001.pdf]

Supplemental materials to the manuscript:

HabitWalk: A Micro-Randomized Trial to Understand and Promote Habit Formation in Physical Activity

Baretta D., Gillmann N., Edgren R., & Inauen J.

Table of Contents

Figure S1: Visual Overview of Study Research Questions 4

Part 1. Supplemental Materials to the Methods Section of the Manuscript 5

Table S1: Sample Characteristics 5

Box S1: Operationalizations of Cue-Behavior Repetition 8

Figure S2: Example of a Possible Randomization of the BCTs 9

Box S2: Instructions Technological Infrastructure 10

Table S2: Missing Values 11

Figure S3: Time Series Characteristics 12

Table S3: Growth Models' Specification 13

Table S4: Multilevel Models' Specification 15

Box S3: Divergence From Registered Analysis 16

Part 2. Supplemental Materials to the Results Section of the Manuscript 17

Table S5: Cue Selection Process in the Analytical Sample 17

Table S6: Summary of Selected Cues 20

Figure S4: Participant's Flow Chart 21

Table S7: Reasons for Quitting the Study 22

Table S8: Predicted Values in Habit Strength 23

Table S9: Model 1 24

Table S10: Model 2 27

Table S11: Model 3 29

Table S12: Sensitivity Analysis After Removing Non-Wearing Days From the Coding of Cue-Behavior Repetition 32

Table S13: Sensitivity Analysis With the Coding of Cue-Behavior Repetition Based on Garmin-Recorded Activities and Daily Evening Reports 34

Table S14: Sensitivity Analysis Including all the Participants who Entered the Intervention Phase....36

Table S15: N-of-1 Level Results for Model 1....37

Figure S5: N-of-1 Level Results for Model 1 - FOREST PLOT....41

Table S16: N-of-1 Level Results for Model 2....42

Figure S6: N-of-1 Level Results for Model 2 – FOREST PLOT....45

Table S17: N-of-1 Level Results for Model 3....46

Figure S7: N-of-1 Level Results for Model 3 – FOREST PLOT....49

Figure S1: Visual Overview of Study Research Questions

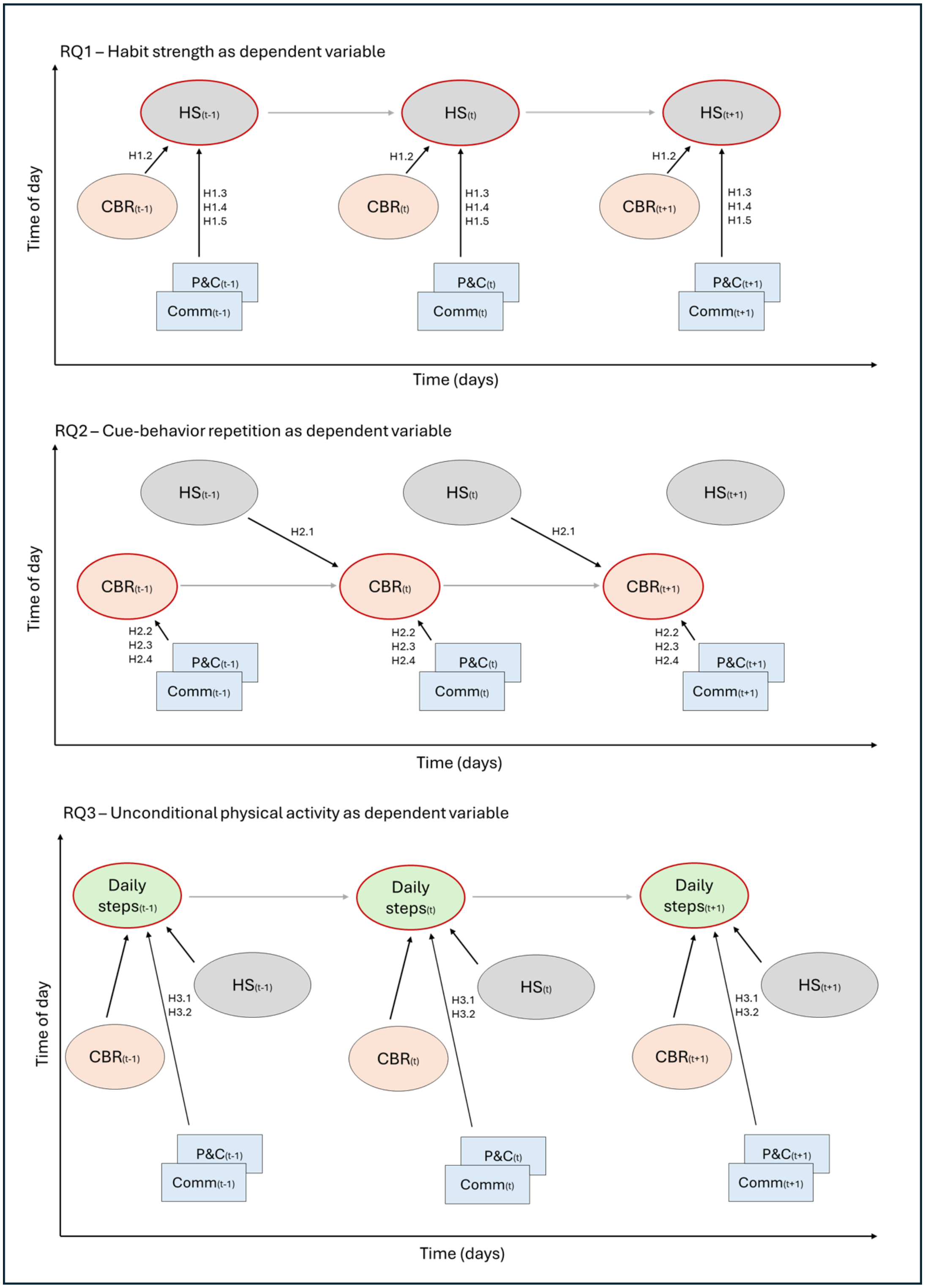

Note. HS = habit strength; CBR = cue-behavior repetition; P&C = BCT prompts and cues; Comm = BCT commitment. The dependent variable is in red circles. Time of day on the y-axis was included to conceptually represent the general sequence of events and measurements as they occurred throughout the day.

Part 1.

Supplemental Materials to the Methods Section of the Manuscript

Table S1: Sample Characteristics

Sample Characteristics and Dropout Analysis

Variable	Overall (n = 45)[1]	Excluded (n = 21)[1]	Included (n = 24)[1]	p-value[2]
Sex				>0.999
Male	4 (8.9%)	2 (9.5%)	2 (8.3%)	
Female	41 (91%)	19 (90%)	22 (92%)	
Age	39 (30, 53)	38 (29, 41)	41 (32, 57)	0.187
BMI	24.2 (21.6, 28.9)	24.2 (21.0, 28.2)	23.9 (22.4, 29.1)	0.850
Unknown	3	0	3	
Civil status				0.800
Single	27 (60%)	12 (57%)	15 (63%)	
Divorced	6 (13%)	3 (14%)	3 (13%)	
In a registered partnership	1 (2.2%)	1 (4.8%)	0 (0%)	
Married	10 (22%)	4 (19%)	6 (25%)	
Widowed	1 (2.2%)	1 (4.8%)	0 (0%)	
Education				0.861
High school diploma	32 (71%)	16 (76%)	16 (67%)	
Completed primary school	1 (2.2%)	0 (0%)	1 (4.2%)	
Completed secondary school	12 (27%)	5 (24%)	7 (29%)	
Higher education				0.628
Completed vocational training (apprenticeship)	9 (20%)	4 (19%)	5 (21%)	
Completed (applied) university degree	27 (60%)	11 (52%)	16 (67%)	
Other educational or vocational qualification	3 (6.7%)	2 (9.5%)	1 (4.2%)	
No educational or vocational training	6 (13%)	4 (19%)	2 (8.3%)	
Employment				0.122
Unemployed	3 (6.7%)	3 (14%)	0 (0%)	
Employed	27 (60%)	9 (43%)	18 (75%)	

Variable	Overall (n = 45)[1]	Excluded (n = 21)[1]	Included (n = 24)[1]	p-value[2]
Homemaker	3 (6.7%)	2 (9.5%)	1 (4.2%)	
Retired	2 (4.4%)	1 (4.8%)	1 (4.2%)	
In training / retraining	10 (22%)	6 (29%)	4 (17%)	
Employment - dichotomous				0.037
Employed	27 (60%)	9 (43%)	18 (75%)	
All other categories	18 (40%)	12 (57%)	6 (25%)	
Monthly net income of the entire household				0.731
Up to 2,000 CHF	2 (4.4%)	2 (9.5%)	0 (0%)	
Btw. 2,001 and 4,000 CHF	3 (6.7%)	2 (9.5%)	1 (4.2%)	
Btw. 4,001 and 6,000 CHF	10 (22%)	5 (24%)	5 (21%)	
Btw. 6,001 and 8,000 CHF	14 (31%)	6 (29%)	8 (33%)	
Btw. 8,001 and 10,000 CHF	6 (13%)	2 (9.5%)	4 (17%)	
More than 10,000 CHF	10 (22%)	4 (19%)	6 (25%)	
Subjective perception of the income				0.273
I do not have enough money to cover my needs.	6 (13%)	4 (19%)	2 (8.3%)	
I have enough money to cover my needs.	33 (73%)	16 (76%)	17 (71%)	
I have more than enough money to cover my needs.	6 (13%)	1 (4.8%)	5 (21%)	
Living situation				0.707
Living by themselves	9 (20%)	5 (24%)	4 (17%)	
Living with family	18 (40%)	8 (38%)	10 (42%)	
Living with partner	13 (29%)	5 (24%)	8 (33%)	
Living in a shared apartment	4 (8.9%)	3 (14%)	1 (4.2%)	
Other	1 (2.2%)	0 (0%)	1 (4.2%)	
Number of other people in the household	2.00 (2.00, 3.00)	2.00 (2.00, 3.00)	2.00 (1.75, 3.00)	0.443
Total MET-minutes/week	1,291 (654, 2,907)	1,032 (742, 2,952)	1,497 (606, 2,907)	0.920
Incomplete data	3	1	2	
MET-minutes/week: Vigorous intensity	720 (350, 1,260)	720 (360, 960)	960 (320, 1,440)	>0.999

Variable	Overall (n = 45)[1]	Excluded (n = 21)[1]	Included (n = 24)[1]	p-value[2]
Incomplete data	21	10	11	
MET-minutes/week: Moderate intensity	480 (240, 900)	240 (150, 570)	570 (390, 960)	0.083
Incomplete data	11	5	6	
MET-minutes/week: Walking	396 (198, 1,056)	330 (198, 1,089)	446 (168, 990)	0.933
Incomplete data	6	2	4	
Categorical score of physical activity				>0.999
Low	11 (24%)	5 (24%)	6 (25%)	
Moderate	31 (69%)	15 (71%)	16 (67%)	
Incomplete data	3 (6.7%)	1 (4.8%)	2 (8.3%)	
Total minutes/day sitting	480 (360, 600)	420 (330, 530)	480 (360, 600)	0.268
Incomplete data	3	2	1	

Note. [1]n (%); Median (IQR); [2]Fisher's exact test for categorical variables; Kruskal-Wallis rank sum test for continuous variables; 'Included' and 'excluded' differentiate between participants who are part of the final analytical sample and those who were excluded based on the frequency of missing values; MET = Metabolic Equivalent of Task; MET-minutes/week = MET level x minutes of activity/day x days per week; MET levels: walking = 3.3 METs, moderate intensity = 4.0 METs, vigorous intensity = 8.0 METs; Low categorical score of physical activity = (a) no activity is reported or (b) some activity is reported but not enough to meet categories 2 (moderate) or 3 (high); Moderate categorical score of physical activity = either of the following 3 criteria: (a) 3 or more days of vigorous activity of at least 20 minutes per day or (b) 5 or more days of moderate-intensity activity and/or walking of at least 30 minutes per day or (c) 5 or more days of any combination of walking, moderate-intensity or vigorous-intensity activities achieving a minimum of at least 600 MET-minutes/week.

Box S1: Operationalizations of Cue-Behavior Repetition

In this study, we conducted a set of sensitivity analysis with diverse operationalizations of cue-behavior repetition as operational definitions might impact the strength of the association between cue-behavior repetition, habit strength and physical activity. We identified 12 different operationalizations of cue-behavior repetition (including the target one offered in the manuscript), resulting from the combination of the following three aspects:

1. **Completion of the target behavior.** Whether the target behavior was only initiated or completed. "Initiated" indicates a walk of at least 1 minute, while "Completed" indicates a walk of at least 15 minutes.
2. **Cadence of the target behavior**. "No constraints" means that the cadence of 70 steps/min was not achieved, while ">70 steps/min" and ">100 steps/min" mean that cadences of 70 and 100 steps/min were achieved, respectively. Even though slower than the scientific definition of brisk walking (100 steps/min), we included also the threshold of 70 steps/min because this cadence is associated with health markers and is more feasible to sustain in free-living contexts (Tudor-Locke et al., 2018).
3. **Proximity to the expected timing of the cue**. Whether the target behavior was recorded at a time aligning with the expected timing of the cue. "No constraints" means there were no time constraints to categorize a recorded walk as an instance of cue-behavior repetition. "Proximal" means that, to be categorized as cue-behavior repetition, a recorded walk should have occurred within two hours before or after the selected cue time.

These are the resulting operational definitions of cue-behavior repetition:

Operational definition	Completion	Cadence	Proximity
CBR.1	*Initiated*	*No constraints*	*No constraints*
CBR.2	Initiated	>70 steps/min	No constraints
CBR.3	Initiated	No constraints	Proximal
CBR.4	Initiated	>70 steps/min	Proximal
CBR.5	Completed	No constraints	No constraints
CBR.6	Completed	>70 steps/min	No constraints
CBR.7	Completed	No constraints	Proximal
CBR.8	Completed	>70 steps/min	Proximal
CBR.9	Initiated	>100 steps/min	No constraints
CBR.10	Initiated	>100 steps/min	Proximal
CBR.11	Completed	>100 steps/min	No constraints
CBR.12	Completed	>100 steps/min	Proximal

Note that CBR.1 is the operationalization we registered and adopted in the main manuscript.

Figure S2: Example of a Possible Randomization of the BCTs

Illustrative Example of a Possible Randomization of the BCTs as Implemented in the Current Study

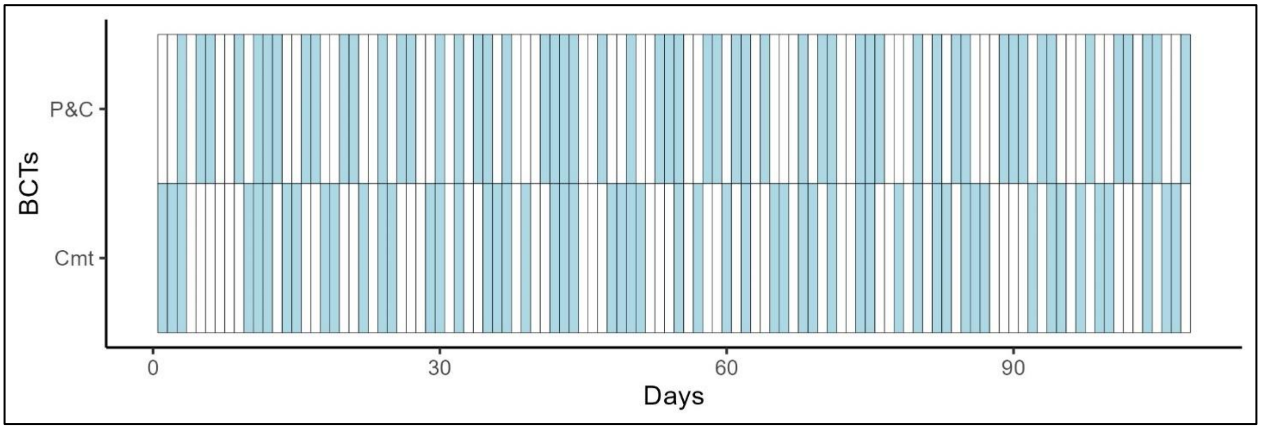

Note. P&C = prompts and cues; Cmt = commitment; Light blue means that the BCT was delivered on that day. The randomization process followed a 6-day urn randomization scheme, ensuring that each BCT was delivered at least three times every six days. This approach aimed to evenly distribute the delivery of each BCT throughout the study, while fully leveraging the randomization design

Box S2: Instructions Technological Infrastructure

Box 2. Instructions received by the participants on how to use the technological infrastructure.

The technological infrastructure: The technological infrastructure for the HabitWalk study was made of the i) the study app (HabitWalk), ii) an activity tracker (Garmin vivosmart 4), and iii) the Fitrockr app (a smartphone app from a technological partner of Garmin that pull the data from the Garmin activity tracker and store it in their internal servers). To ensure the proper functioning of the Fitrockr app, it was necessary to ensure that the Garmin Connect app was not installed, as per Fitrockr's recommendations.

Instructions: Participants were advised to consistently wear the activity tracker on the same non-dominant hand throughout the day. It was optional for participants to wear it during the night. If desired, they were provided with a sheet containing instructions. They were informed that the activity tracker would record their daily steps and would require regular synchronization with the app. Furthermore, participants were guided on how to synchronize the Garmin device with the Fitrockr Hub app and how to charge it. Additionally, they were instructed on initiating and saving a specific activity (15-minute brisk walk), emphasizing that this should only be recorded when the walk occurred after encountering their cue. Regarding the HabitWalk app, participants were shown where they could fill out the questionnaires, access their if-then plans, and find the instruction videos for the activity tracker within the app. Lastly, participants received an overview of the HabitWalk study and an estimate of the approximate time different tasks would take.

Table S2: Missing Values

Number of Missing Values for Each Time Serial Variable

Variable	Overall (*n* = 40)	Excluded (*n* = 16)	Included (*n* = 24)
	M (%)	*M* (%)	*M* (%)
	Range (%)	Range (%)	Range (%)
Habit strength			
105 days	50.1 (47.7%)	89.6 (85.3%)	23.8 (22.7%)
	2–105 (1.9–100%)	73–105 (69.5–100%)	2–67 (1.9–63.8%)
66 days[1]	27.2 (41.2%)	52.4 (79.4%)	10.4 (15.7%)
	0–66 (0–100%)	34–66 (51.5–100%)	0–32 (0–48.5%)
Cue-behavior repetition			
105 days	60.8 (57.9%)	89.3 (85.0%)	41.9 (39.9%)
	5–105 (4.8–100%)	29–105 (27.6–100%)	5–102 (4.8–97.1%)
Daily steps			
105 days	43.9 (41.8%)	73.3 (69.8%)	24.3 (23.1%)
	4–105 (3.8–100%)	22–105 (21.0–100%)	4–89 (3.8–84.8%)

Note. ‘Included' and 'Excluded' differentiate between participants who are part of the final analytical sample and those who were excluded based on the frequency of missing values.

[1] Statistics calculated out of the first 66 days of the experimental phase.

Figure S3: Time Series Characteristics

Time Series Characteristics and Missing Values in Habit Strength

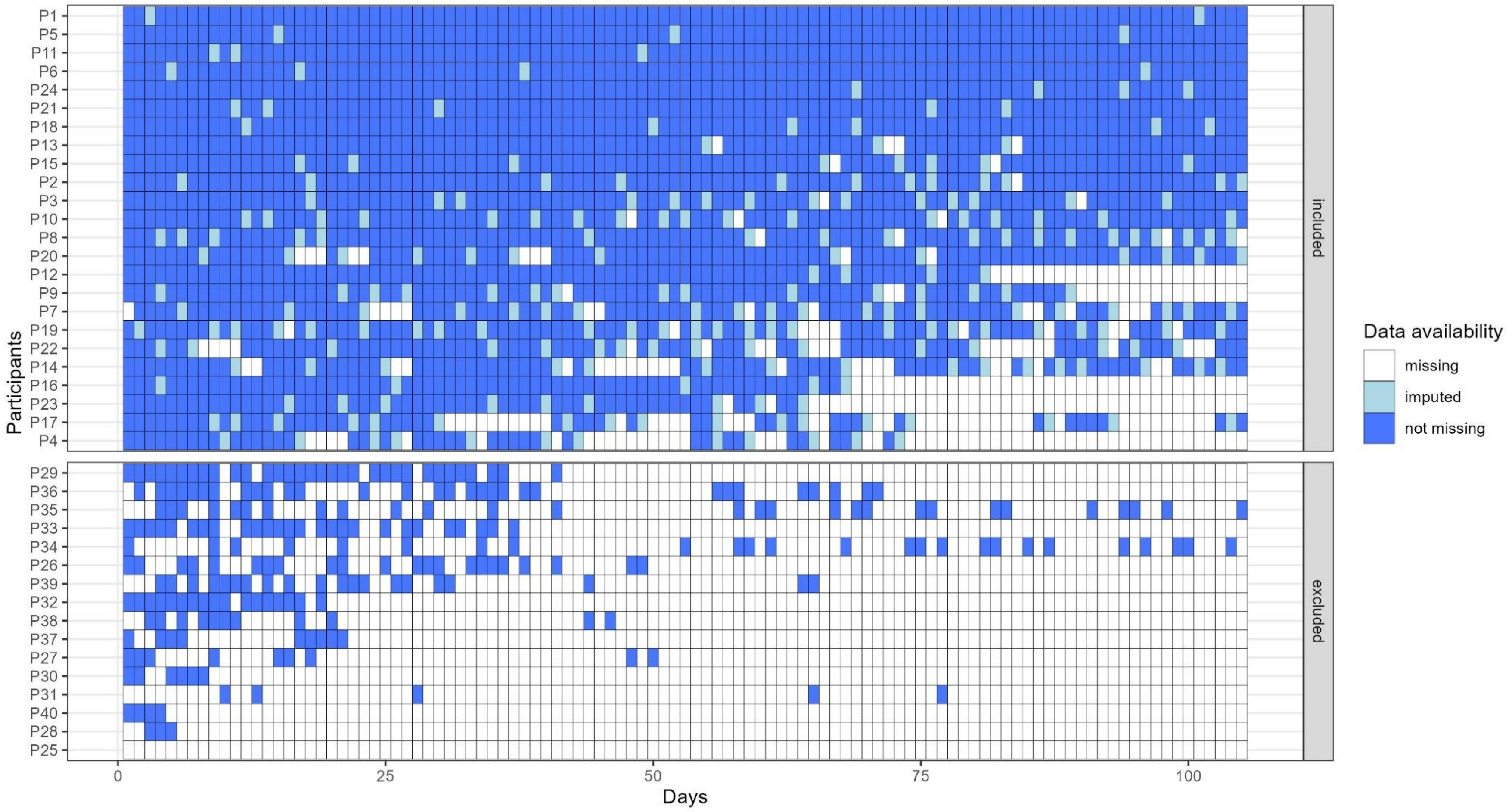

Note. 'Included' and 'Excluded' differentiate between participants who are part of the final analytical sample and those who were excluded based on the frequency of missing values.

Table S3: Growth Models' Specification

Growth Models' Specification

Model	Specification
**Constant**	
Formula	HS = $\beta 0$
R code	lm(HS ~ 1, data)
**Linear**	
Formula	HS = $\beta 0$ + $\beta 1$(time), where $\beta 1$ representing the linear effect of time.
R code	lm(HS ~ Time, data)
**Quadratic**	
Formula	HS = $\beta 0$ + $\beta 1$(time) + $\beta 2$(time)^2 + ε, where $\beta 1$ and $\beta 2$ representing the linear and quadratic effect of time respectively.
R code	lm(HS ~ Time + I(Time^2), data)
**Asymptotic**	
Formula	HS = $\beta 0 + ((\beta 1 - \beta 0) * \exp(-\exp(\beta 2) * Time)) + \varepsilon$, where $\beta 1$ representing the response on day zero, $\beta 0$ representing the horizontal asymptote on the right side, $\beta 2$ representing the natural logarithm of the rate constant.
R code	nls_multstart (HS ~ SSasymp(Time, Asym, R0, lrc), data, iter = 5000, start_lower = c(Asym=0, R0=1, lrc=1), start_upper = c(Asym=4, R0=4, lrc=5), supp_errors = 'Y', na.action = na.omit, lower = c(Asym=1, R0=0.5, lrc=-Inf), upper = c(Asym=5, R0=5, lrc=Inf), control = nls.lm.control(maxfev = 1000, maxiter = 200))

GAM	
Formula	HS = β0 + f(Time) + ε, where f represents a one-dimensional smoother term for the variable time to discover its functional forms. The number of smooth terms will be defined exploratively based on the data and length of the time series.
R code	gam(HS ~ s(Time, bs = 'tp', k = round(105/7)), method = 'REML', data)

Note. HS = habit strength; the syntax of the asymptotic model is adapted from Keller et al. (2021a).

Table S4: Multilevel Models' Specification

Model Specification and Selection for Main Analyses

Model specification	BIC	AIC
Model 1		
HS_t ~ Time + HS_{t-1} + CBR_t + Com_t * $P\&C_t$ + $P\&C_t$ * Time + Com_t * Time + (1 | ID)	1831.3	1769.1
HS_t ~ Time + HS_{t-1} + CBR_t + Com_t * $P\&C_t$ + $P\&C_t$ * Time + Com_t * Time + (Time | ID)	1702.7	1629.2
HS_t ~ Time + HS_{t-1} + CBR_t + Com_t * $P\&C_t$ + $P\&C_t$ * Time + Com_t * Time + (Time + CBR_t | ID)	1644.7	1554.2
**HS_t ~ Time + HS_{t-1} + CBR_t + Com_t * $P\&C_t$ + $P\&C_t$ * Time + Com_t * Time + (Time + CBR_t + HS_{t-1} || ID)**	**1479.8**	**1400.6**
Model 2		
CBR_t ~ Time + CBR_{t-1} + HS_{t-1} + Com_t * $P\&C_t$ + (1 | ID)	2405.3	2359.8
**CBR_t ~ Time + CBR_{t-1} + HS_{t-1} + Com_t * $P\&C_t$ + (Time || ID)**	**2401.4**	**2350.2**
CBR_t ~ Time + CBR_{t-1} + HS_{t-1} + Com_t * $P\&C_t$ + (Time + HS_{t-1} || ID)	2408.6	2351.7
CBR_t ~ Time + CBR_{t-1} + HS_{t-1} + Com_t * $P\&C_t$ + (Time + CBR_{t-1} || ID)	2408.8	2351.9
CBR_t ~ Time + CBR_{t-1} + HS_{t-1} + Com_t * $P\&C_t$ + (Time + HS_{t-1} + $P\&C_t$ + Com_t || ID)	2422.1	2353.8
Model 3		
**$Steps_t$ ~ Time + $Steps_{t-1}$ + HS_t + CBR_t + Com_t * $P\&C_t$ + (1 | ID)**	**33627**	**33572**
$Steps_t$ ~ Time + $Steps_{t-1}$ + HS_t + CBR_t + Com_t * $P\&C_t$ + (Time | ID)	33637	33571
$Steps_t$ ~ Time + $Steps_{t-1}$ + HS_t + CBR_t + Com_t * $P\&C_t$ + (HS_t | ID)	33641	33576
$Steps_t$ ~ Time + $Steps_{t-1}$ + HS_t + CBR_t + Com_t * $P\&C_t$ + (CBR_t | ID)	33638	33572

Note. Models' formula is specified using the syntax from the *lme4* R package. The final selected model is in bold. ~ = to be read as 'explained by'. Random effects: A single vertical bar indicates correlated random intercept and slope, while a double vertical bar indicates uncorrelated random intercept and slope; HS = habit strength; CBR = cue-behavior repetition; Com = BCT commitment; P&C = BCT prompts and cues; ID = participant identifier; AIC = Akaike Information Criterion; BIC = Bayesian Information Criterion. We also tested more complex models, which we did not report here because they led to singular fit or converge failure.

Box S3: Divergence From Registered Analysis

First, as outlined in the co-registration, our plan encompassed both idiographic (i.e., n-of-1) and nomothetic (i.e., multilevel modeling) analyses to test our hypotheses, with a primary focus on the former approach. However, due to a higher-than-anticipated rate of missing values, we chose to test our hypotheses preferring the multilevel approach, given its greater flexibility in handling missing data (Stadnitski & Wild, 2019). Nonetheless, as part of the sensitivity analysis, we still analyzed a subset of participants with high data quality (i.e., less than 20% missing values in habit strength time series over the first 66 intervention days) using the idiographic n-of-1 approach.

Second, regarding H1.1 concerning the shape of habit formation, we omitted assessing the constant and linear models in our registration as we assumed a non-linear growth (see Gardner et al., 2022). However, we eventually decided to include them after visually inspecting the time series and noticing that simpler models represented a better fit.

Part 2.

Supplemental Materials to the Results Section of the Manuscript

Table S5: Cue Selection Process in the Analytical Sample

Participant	Day 1	Day 2	Day 3	Day 4	Day 5	Day 6	Final cue (Day 6)	First HS value
**P8**	After dinner	After dinner	After breakfast	After lunch	-	-	After dinner	3.75
**P24**	After work	-	-	After dinner	-	-	After dinner	3.75
**P14**	After work	-	-	-	-	-	After work	3.50
**P11**	After dinner	After work	-	-	-	-	After dinner	3.25
**P18**	After lunch	-	-	-	-	-	After lunch	3.25
**P2**	After 5 p.m.	-	-	-	-	After 4 p.m.	At 5 p.m.	2.75
**P1**	After phone with X	After work (just not every day)	-	-	-	-	After phone with X	2.00
**P6**	After breakfast	After breakfast, after reading the newspaper and after showering: at 8.30.	-	Between 8.30 and 9.30 a.m.	... and after meditating	-	Between 8.30 and 9.30, after breakfast, after showering and after meditating	2.00
**P10**	After dinner	-	After dish washing in the evening	-	-	-	After dinner	2.00
**P15**	-	Before dinner	After brushing	-	-	After returning	After dinner	2.00

Participant	Day 1	Day 2	Day 3	Day 4	Day 5	Day 6	Final cue (Day 6)	First HS value
			teeth's morning			home from university		
**P17**	After lunch	after the band rehearsal/ going out	at the weekend after breakfast	After work	After breakfast	At coffee break time in the morning or afternoon	After lunch	2.00
**P7**	After work	After work	-	After coffee in the morning	-	After lunch	After work	1.50
**P3**	After work	-	-	-	-	Walking to work in the morning	After work	1.25
**P22**	After dinner	-	After work	-	-	-	After dinner	1.25
**P4**	After snack in the afternoon	-	-	At 6.00 p.m.	-	After snack in the afternoon	After snack in the afternoon	1.00
**P5**	After brushing teeth	-	After brushing teeth morning	-	-	-	After brushing teeth morning	1.00
**P9**	After lunch	-	After dinner	-	-	-	After lunch	1.00
**P12**	After grinding coffee in the morning	-	In the morning after first cup of coffee, between medication and breakfast, without dog	After brushing teeth evening	-	After medication and 1st cup of coffee at 7.45 am without dog	After medication and 1st cup of coffee at 7.45 am without dog	1.00

Participant	Day 1	Day 2	Day 3	Day 4	Day 5	Day 6	Final cue (Day 6)	First HS value
**P13**	After snack in the afternoon	After dinner	-	After coffee in the morning	-	-	After snack in the afternoon	1.00
**P16**	After breakfast	After dinner	-	After studying in the evening	After waking up in the morning	-	After brushing your teeth's morning	1.00
**P19**	After breakfast	-	-	-	-	-	After breakfast	1.00
**P20**	After lunch	After coffee break in the afternoon	After work	-	-	-	After lunch	1.00
**P21**	After dinner	Before dinner	On the way home (get off one stop earlier)	-	-	-	After dinner	1.00
**P23**	After work	-	After lunch	-	-	-	After lunch	1.00

Note. First HS (habit strength) value is from the observed data. Participants are sorted in ascending order based on the first HS value.

Table S6: Summary of Selected Cues

Summary of the Selected Cues

Selected cue	Participants		
	All (n = 40)	Included (n = 24)	Excluded (n = 16)
After breakfast	5 (12.5%)	3 (12.5%)	2 (12.5%)
After brushing teeth morning	3 (7.5%)	2 (8.3%)	1 (6.2%)
After lunch	9 (22.5%)	5 (20.8%)	4 (25%)
After snack in the afternoon	2 (5%)	2 (8.3%)	0 (0%)
After work	4 (10%)	3 (12.5%)	1 (6.2%)
After dinner	11 (27.5%)	7 (29.2%)	4 (25%)
Other	6 (15%)	2 (8.3%)	4 (25%)

Note. ‘Included' and 'Excluded' differentiate between participants who are part of the final analytical sample and those who were excluded based on the frequency of missing values. The list of the selected cues is based on a categorization done by the authors.

Figure S4: Participant's Flow Chart

Participant's Flow Chart

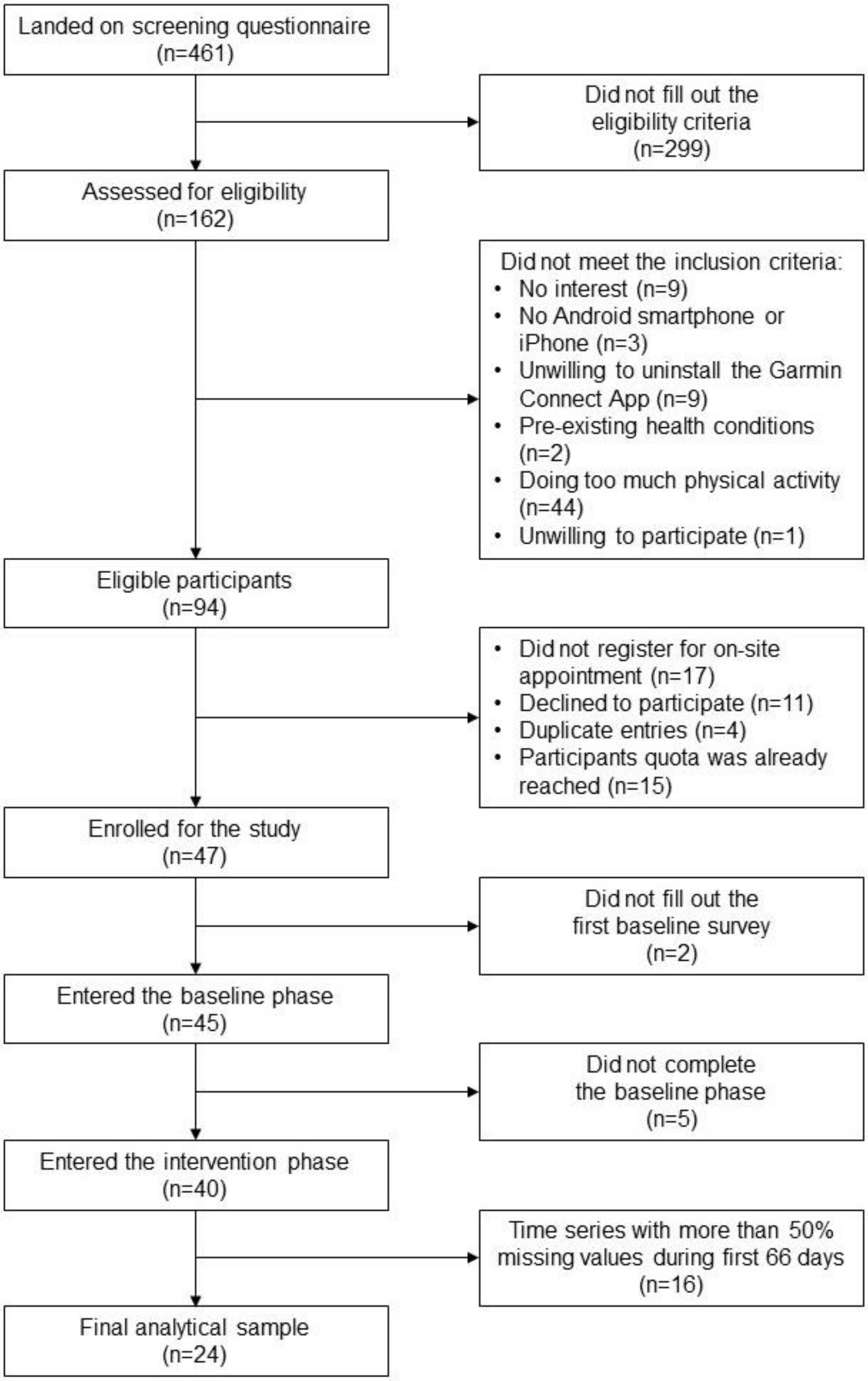

Table S7: Reasons for Quitting the Study

Overview of Reasons why Participants Decided to Formally Withdraw From the Study

Participant	Day of quitting	Reason
Included participants		
P23	67	No reason given
P16	74	Felt too much pressure and was too stressed, chose the wrong cue
P12	81	No reason given
Excluded participants		
P25	4	Chronic health issue returned and makes it very hard to keep a routine like walking at the same time everyday when he cannot predict the chronic pain
P40	5	Problems with technology, the time involved is too much
P30	9	No reason given
P28	9	Too much stressed and nervous, push notifications intrusive, synchronization did not work
P29	42	No reason given
P32	44	Said that she is too inconsistent and forgetful
P26	54	No reason given
P27	63	Felt not mentally well, not much strength and energy, and problems with the smartphone
P39	76	Felt too stressed, has a lot of other administrative tasks

Table S8: Predicted Values in Habit Strength

First and Last Predicted Values in Habit Strength and Their Difference Score for Each Participant

Participant	Best model	First observed value	First predicted value	Last predicted value	Difference between first and last predicted value	Frequency of CBR	Random effect of CBR from MLM 1
P24	linear	3.75	3.803	4.498	0.695	86	0.229
P11	constant	3.25	3.957	3.957	0.000	84	0.215
P21	GAM	1.00	1.524	3.648	2.124	93	0.128
P8	GAM	3.75	3.757	3.516	-0.241	80	0.035
P7	asymptotic	1.50*	1.155	3.376	2.221	80	0.120
P14	linear	3.50	3.526	3.105	-0.421	73	0.008
P12	linear	1.00	0.908	3.055	2.147	70	0.087
P18	asymptotic	3.25	3.483	2.833	-0.650	59	0.230
P15	quadratic	2.00	1.795	2.816	1.021	93	0.050
P1	GAM	2.00	1.974	2.566	0.592	100	0.148
P20	linear	1.00	1.238	2.555	1.317	58	0.033
P6	linear	2.00	2.414	2.039	-0.375	95	0.028
P17	constant	2.00	2.024	2.024	0.000	3	0.082
P2	GAM	2.75	2.911	1.986	-0.925	52	0.169
P3	GAM	1.25	1.429	1.973	0.544	35	0.717
P10	GAM	2.00	2.045	1.957	-0.088	63	0.102
P22	GAM	1.25	1.605	1.883	0.278	78	0.014
P19	GAM	1.00	1.512	1.840	0.328	77	0.229
P9	linear	1.00	1.293	1.736	0.443	43	0.242
P4	linear	1.00	0.964	1.551	0.587	19	0.121
P13	constant	1.00	1.282	1.282	0.000	36	0.004
P23	GAM	1.00	0.941	1.126	0.185	25	0.161
P5	constant	1.00	1.123	1.123	0.000	52	0.281
P16	quadratic	1.00	1.161	-2.206	-3.367	48	0.323

Note. *Value was observed on the second day of the experimental phase due to a missing value on the first day; Frequency of CBR = total number of cue-behavior repetitions (CBR) that participants performed during the experimental phase. GAM = Generalized Additive Models. Participants are sorted in descending order based on the last predicted value.

Table S9: Model 1

Model 1 (Dependent Variable is Habit Strength) Under Different Operationalizations of Cue-Behavior Repetition

Variable		CBR.1	CBR.2	CBR.3	CBR.4	CBR.5	CBR.6	CBR.7	CBR.8	CBR.9	CBR.10	CBR.11	CBR.12
Intercept	Est.	**2.16**	**2.19**	**2.21**	**2.22**	**2.17**	**2.19**	**2.21**	**2.22**	**2.24**	**2.25**	**2.25**	**2.25**
	(SE)	**(0.19)**	**(0.19)**	**(0.19)**	**(0.19)**	**(0.19)**	**(0.19)**	**(0.18)**	**(0.19)**	**(0.19)**	**(0.19)**	**(0.19)**	**(0.19)**
	95% CI	**[1.79, 2.54]**	**[1.81, 2.57]**	**[1.84, 2.58]**	**[1.85, 2.59]**	**[1.80, 2.55]**	**[1.82, 2.57]**	**[1.84, 2.58]**	**[1.85, 2.59]**	**[1.87, 2.62]**	**[1.87, 2.62]**	**[1.87, 2.63]**	**[1.87, 2.62]**
	β	**-0.03**	**-0.04**	**-0.04**	**-0.04**	**-0.03**	**-0.04**	**-0.04**	**-0.04**	**-0.05**	**-0.05**	**-0.05**	**-0.04**
	(SE)	**(0.18)**	**(0.18)**	**(0.18)**	**(0.18)**	**(0.18)**	**(0.18)**	**(0.18)**	**(0.18)**	**(0.18)**	**(0.18)**	**(0.18)**	**(0.18)**
Time	Est.	**<0.01**	**<0.01**	**<0.01**	**<0.01**	**<0.01**	**<0.01**	**<0.01**	**<0.01**	**<0.01**	**<0.01**	**<0.01**	**<0.01**
	(SE)	**(0.00)**	**(0.00)**	**(0.00)**	**(0.00)**	**(0.00)**	**(0.00)**	**(0.00)**	**(0.00)**	**(0.00)**	**(0.00)**	**(0.00)**	**(0.00)**
	95% CI	**[0.00, 0.00]**	**[0.00, 0.00]**	**[0.00, 0.00]**	**[0.00, 0.00]**	**[0.00, 0.00]**	**[0.00, 0.00]**	**[0.00, 0.00]**	**[0.00, 0.00]**	**[0.00, 0.00]**	**[0.00, 0.00]**	**[0.00, 0.00]**	**[0.00, 0.00]**
	β	**0.07**	**0.08**	**0.07**	**0.07**	**0.08**	**0.08**	**0.07**	**0.07**	**0.06**	**0.06**	**0.06**	**0.06**
	(SE)	**(0.03)**	**(0.03)**	**(0.03)**	**(0.03)**	**(0.03)**	**(0.03)**	**(0.03)**	**(0.03)**	**(0.03)**	**(0.03)**	**(0.03)**	**(0.03)**
HS $_{t-1}$	Est.	**0.35**	**0.36**	**0.36**	**0.36**	**0.35**	**0.36**	**0.36**	**0.36**	**0.35**	**0.35**	**0.36**	**0.36**
	(SE)	**(0.06)**	**(0.06)**	**(0.06)**	**(0.06)**	**(0.06)**	**(0.06)**	**(0.06)**	**(0.06)**	**(0.06)**	**(0.06)**	**(0.06)**	**(0.06)**
	95% CI	**[0.23, 0.46]**	**[0.24, 0.47]**	**[0.24, 0.47]**	**[0.24, 0.47]**	**[0.23, 0.47]**	**[0.24, 0.48]**	**[0.24, 0.47]**	**[0.24, 0.48]**	**[0.23, 0.47]**	**[0.24, 0.47]**	**[0.24, 0.48]**	**[0.24, 0.47]**
	β	**0.16**	**0.16**	**0.16**	**0.16**	**0.16**	**0.16**	**0.16**	**0.17**	**0.16**	**0.16**	**0.16**	**0.16**
	(SE)	**(0.03)**	**(0.03)**	**(0.03)**	**(0.03)**	**(0.03)**	**(0.03)**	**(0.03)**	**(0.03)**	**(0.03)**	**(0.03)**	**(0.03)**	**(0.03)**
CBR $_{t}$	Est.	**0.16**	**0.14**	**0.12**	**0.13**	**0.15**	**0.15**	**0.12**	**0.14**	**0.09**	**0.11**	0.09	**0.12**
	(SE)	**(0.04)**	**(0.04)**	**(0.04)**	**(0.04)**	**(0.04)**	**(0.04)**	**(0.04)**	**(0.04)**	**(0.04)**	**(0.05)**	(0.05)	**(0.05)**
	95% CI	**[0.08, 0.23]**	**[0.07, 0.21]**	**[0.04, 0.20]**	**[0.05, 0.20]**	**[0.07, 0.22]**	**[0.07, 0.22]**	**[0.05, 0.20]**	**[0.06, 0.21]**	**[0.00, 0.18]**	**[0.01, 0.20]**	[-0.01, 0.19]	**[0.02, 0.21]**
	β	**0.08**	**0.07**	**0.06**	**0.06**	**0.08**	**0.07**	**0.06**	**0.07**	**0.04**	**0.04**	0.04	**0.04**
	(SE)	**(0.02)**	**(0.02)**	**(0.02)**	**(0.02)**	**(0.02)**	**(0.02)**	**(0.02)**	**(0.02)**	**(0.02)**	**(0.02)**	(0.02)	**(0.02)**
P&C $_{t}$	Est.	-0.05	-0.05	-0.04	-0.05	-0.05	-0.05	-0.04	-0.04	-0.04	-0.04	-0.04	-0.04
	(SE)	(0.03)	(0.03)	(0.03)	(0.03)	(0.03)	(0.03)	(0.03)	(0.03)	(0.03)	(0.03)	(0.03)	(0.03)

Variable		CBR.1	CBR.2	CBR.3	CBR.4	CBR.5	CBR.6	CBR.7	CBR.8	CBR.9	CBR.10	CBR.11	CBR.12
	95% CI	[-0.11, 0.00]	[-0.11, 0.01]	[-0.10, 0.02]	[-0.11, 0.01]	[-0.11, 0.01]	[-0.11, 0.01]	[-0.10, 0.02]	[-0.10, 0.02]	[-0.10, 0.02]	[-0.10, 0.02]	[-0.10, 0.02]	[-0.10, 0.02]
	β	<0.01	<0.01	<0.01	<0.01	<0.01	<0.01	<0.01	<0.01	<0.01	<0.01	<0.01	<0.01
	(SE)	(0.01)	(0.01)	(0.01)	(0.01)	(0.01)	(0.01)	(0.01)	(0.01)	(0.01)	(0.01)	(0.01)	(0.01)
Commitment $_t$	Est.	-0.03	-0.03	-0.03	-0.04	-0.03	-0.04	-0.03	-0.04	-0.03	-0.03	-0.03	-0.03
	(SE)	(0.03)	(0.03)	(0.03)	(0.03)	(0.03)	(0.03)	(0.03)	(0.03)	(0.03)	(0.03)	(0.03)	(0.03)
	95% CI	[-0.09, 0.03]	[-0.09, 0.03]	[-0.09, 0.03]	[-0.10, 0.02]	[-0.09, 0.03]	[-0.10, 0.02]	[-0.09, 0.03]	[-0.10, 0.02]	[-0.09, 0.03]	[-0.09, 0.03]	[-0.09, 0.03]	[-0.09, 0.03]
	β	<0.01	<0.01	<0.01	<0.01	<0.01	<0.01	<0.01	<0.01	<0.01	<0.01	<0.01	<0.01
	(SE)	(0.01)	(0.01)	(0.01)	(0.01)	(0.01)	(0.01)	(0.01)	(0.01)	(0.01)	(0.01)	(0.01)	(0.01)
P&C $_t$: Commitment $_t$	Est.	**0.06**	**0.06**	**0.06**	**0.05**	**0.05**	**0.05**	**0.05**	0.05	0.05	0.05	0.04	0.04
	(SE)	**(0.03)**	**(0.03)**	**(0.03)**	**(0.03)**	**(0.03)**	**(0.03)**	**(0.03)**	(0.03)	(0.03)	(0.03)	(0.03)	(0.03)
	95% CI	**[0.00, 0.11]**	**[0.00, 0.11]**	**[0.00, 0.11]**	**[0.00, 0.11]**	**[0.00, 0.11]**	**[0.00, 0.11]**	**[0.00, 0.11]**	[-0.01, 0.11]	[-0.01, 0.10]	[-0.01, 0.10]	[-0.01, 0.10]	[-0.01, 0.10]
	β	**0.01**	**0.01**	**0.01**	**0.01**	**0.01**	**0.01**	**0.01**	0.01	0.01	0.01	0.01	0.01
	(SE)	**(0.01)**	**(0.01)**	**(0.01)**	**(0.01)**	**(0.01)**	**(0.01)**	**(0.01)**	(0.01)	(0.01)	(0.01)	(0.01)	(0.01)
Time: P&C $_t$	Est.	<0.01	<0.01	<0.01	<0.01	<0.01	<0.01	<0.01	<0.01	<0.01	<0.01	<0.01	<0.01
	(SE)	(0.00)	(0.00)	(0.00)	(0.00)	(0.00)	(0.00)	(0.00)	(0.00)	(0.00)	(0.00)	(0.00)	(0.00)
	95% CI	[0.00, 0.00]	[0.00, 0.00]	[0.00, 0.00]	[0.00, 0.00]	[0.00, 0.00]	[0.00, 0.00]	[0.00, 0.00]	[0.00, 0.00]	[0.00, 0.00]	[0.00, 0.00]	[0.00, 0.00]	[0.00, 0.00]
	β	0.01	0.01	<0.01	0.01	0.01	0.01	<0.01	0.01	<0.01	<0.01	0.01	<0.01
	(SE)	(0.01)	(0.01)	(0.01)	(0.01)	(0.01)	(0.01)	(0.01)	(0.01)	(0.01)	(0.01)	(0.01)	(0.01)
Time: Commitment $_t$	Est.	<0.01	<0.01	<0.01	<0.01	<0.01	<0.01	<0.01	<0.01	<0.01	<0.01	<0.01	<0.01
	(SE)	(0.00)	(0.00)	(0.00)	(0.00)	(0.00)	(0.00)	(0.00)	(0.00)	(0.00)	(0.00)	(0.00)	(0.00)
	95% CI	[0.00, 0.00]	[0.00, 0.00]	[0.00, 0.00]	[0.00, 0.00]	[0.00, 0.00]	[0.00, 0.00]	[0.00, 0.00]	[0.00, 0.00]	[0.00, 0.00]	[0.00, 0.00]	[0.00, 0.00]	[0.00, 0.00]
	β	<0.01	<0.01	<0.01	<0.01	<0.01	<0.01	<0.01	<0.01	<0.01	<0.01	<0.01	<0.01
	(SE)	(0.01)	(0.01)	(0.01)	(0.01)	(0.01)	(0.01)	(0.01)	(0.01)	(0.01)	(0.01)	(0.01)	(0.01)
SD (Intercept id)		0.91	0.92	0.90	0.90	0.91	0.92	0.90	0.90	0.92	0.92	0.92	0.91
SD (Time id)		<0.01	<0.01	<0.01	<0.01	<0.01	<0.01	<0.01	<0.01	<0.01	<0.01	<0.01	<0.01

Variable	CBR.1	CBR.2	CBR.3	CBR.4	CBR.5	CBR.6	CBR.7	CBR.8	CBR.9	CBR.10	CBR.11	CBR.12
SD (CBR id)	0.17	0.15	0.17	0.15	0.16	0.15	0.17	0.16	0.18	0.17	0.19	0.18
SD (HS id)	0.25	0.25	0.25	0.25	0.25	0.25	0.25	0.25	0.26	0.25	0.26	0.26
SD (Observations)	0.31	0.32	0.32	0.32	0.31	0.32	0.32	0.32	0.32	0.32	0.32	0.32
Num.Obs.	2107	2107	2107	2107	2107	2107	2107	2107	2107	2107	2107	2107
R2 Marg.	0.041	0.040	0.040	0.040	0.041	0.041	0.040	0.040	0.035	0.035	0.035	0.036
R2 Cond.	0.900	0.898	0.894	0.894	0.898	0.898	0.893	0.893	0.895	0.894	0.895	0.894
AIC	1468.5	1520.5	1515.4	1542.9	1493.9	1525.2	1527.2	1547.8	1576.6	1579.5	1580.8	1583.2
BIC	1547.6	1599.7	1594.5	1622.1	1573.1	1604.4	1606.3	1627.0	1655.8	1658.7	1659.9	1662.4
ICC	0.9	0.9	0.9	0.9	0.9	0.9	0.9	0.9	0.9	0.9	0.9	0.9
RMSE	0.31	0.31	0.31	0.31	0.31	0.31	0.31	0.31	0.31	0.32	0.31	0.32

Note. CBR.1 is the operationalization of cue-behavior repetition as done in the main manuscript. The others reflect the labels and definitions provided in Box 1; Significant fixed effects (p-value < .05) are in bold; β = standardized effect size; HS = habit strength; CBR = cue-behavior repetition; P&C = prompts and cues.

Table S10: Model 2

Model 2 (Dependent Variable is Cue-Behavior Repetition) Under Different Operationalizations of Cue-Behavior Repetition

Variable		CBR.1	CBR.2	CBR.3	CBR.4	CBR.5	CBR.6	CBR.7	CBR.8	CBR.9	CBR.10	CBR.11	CBR.12
Intercept	OR	**2.64**	1.71	1.26	1.03	**2.09**	1.36	1.11	0.88	**0.39**	**0.27**	**0.32**	**0.22**
	(SE)	**(0.78)**	(0.53)	(0.32)	(0.29)	**(0.56)**	(0.40)	(0.28)	(0.24)	**(0.14)**	**(0.10)**	**(0.12)**	**(0.08)**
	95%	**[1.49,**	[0.92,	[0.76,	[0.58,	**[1.23,**	[0.75,	[0.67,	[0.51,	**[0.18,**	**[0.12,**	**[0.15,**	**[0.10,**
	CI	**4.86]**	3.21]	2.10]	1.80]	**3.62]**	2.46]	1.84]	1.52]	**0.79]**	**0.56]**	**0.65]**	**0.45]**
	β	**1.92**	0.86	0.69	0.38	**1.33**	0.66	0.55	0.31	**0.17**	**0.09**	**0.14**	**0.08**
	(SE)	**(0.50)**	(0.24)	(0.17)	(0.10)	**(0.33)**	(0.18)	(0.14)	(0.09)	**(0.06)**	**(0.03)**	**(0.05)**	**(0.03)**
Time	OR	**0.99**	**0.98**	**0.99**	**0.98**	**0.99**	**0.98**	**0.98**	**0.98**	**0.98**	**0.98**	**0.98**	**0.98**
	(SE)	**(0.00)**	**(0.00)**	**(0.00)**	**(0.00)**	**(0.00)**	**(0.00)**	**(0.00)**	**(0.00)**	**(0.00)**	**(0.00)**	**(0.00)**	**(0.00)**
	95%	**[0.98,**	**[0.98,**	**[0.98,**	**[0.97,**	**[0.98,**	**[0.97,**	**[0.97,**	**[0.97,**	**[0.98,**	**[0.97,**	**[0.98,**	**[0.97,**
	CI	**0.99]**	**0.99]**	**0.99]**	**0.99]**	**0.99]**	**0.99]**	**0.99]**	**0.99]**	**0.99]**	**0.99]**	**0.99]**	**0.99]**
	β	**0.66**	**0.61**	**0.68**	**0.59**	**0.66**	**0.60**	**0.65**	**0.57**	**0.65**	**0.62**	**0.66**	**0.64**
	(SE)	**(0.06)**	**(0.06)**	**(0.07)**	**(0.07)**	**(0.06)**	**(0.06)**	**(0.08)**	**(0.07)**	**(0.05)**	**(0.07)**	**(0.06)**	**(0.07)**
CBR $_{t-1}$	OR	**1.52**	**1.41**	1.20	1.09	1.24	**1.35**	1.05	1.01	**1.42**	0.97	**1.49**	1.05
	(SE)	**(0.18)**	**(0.16)**	(0.13)	(0.13)	(0.14)	**(0.15)**	(0.12)	(0.12)	**(0.19)**	(0.15)	**(0.20)**	(0.16)
	95%	**[1.20,**	**[1.13,**	[0.97,	[0.86,	[0.99,	**[1.08,**	[0.85,	[0.80,	**[1.10,**	[0.72,	**[1.15,**	[0.78,
	CI	**1.92]**	**1.77]**	1.48]	1.37]	1.55]	**1.68]**	1.31]	1.28]	**1.84]**	1.30]	**1.94]**	1.42]
	β	**1.22**	**1.19**	1.10	1.04	1.11	**1.16**	1.03	1.00	**1.17**	0.99	**1.19**	1.02
	(SE)	**(0.07)**	**(0.07)**	(0.06)	(0.06)	(0.06)	**(0.07)**	(0.06)	(0.06)	**(0.07)**	(0.06)	**(0.07)**	(0.06)
HS $_{t-1}$	OR	1.13	1.07	1.06	1.05	1.12	1.01	1.02	1.01	1.11	1.08	1.01	0.98
	(SE)	(0.14)	(0.13)	(0.13)	(0.13)	(0.14)	(0.12)	(0.13)	(0.13)	(0.15)	(0.16)	(0.14)	(0.15)
	95%	[0.88,	[0.84,	[0.84,	[0.82,	[0.88,	[0.79,	[0.80,	[0.78,	[0.85,	[0.81,	[0.76,	[0.72,
	CI	1.45]	1.36]	1.35]	1.36]	1.43]	1.28]	1.30]	1.30]	1.45]	1.46]	1.33]	1.32]
	β	1.06	1.04	1.03	1.03	1.06	1.01	1.01	1.01	1.05	1.04	1.01	1.00
	(SE)	(0.06)	(0.06)	(0.06)	(0.06)	(0.06)	(0.06)	(0.06)	(0.06)	(0.06)	(0.07)	(0.06)	(0.07)
P&C $_t$	OR	1.08	1.04	0.92	0.90	1.07	1.03	0.95	0.92	0.81	0.76	0.85	0.76
	(SE)	(0.16)	(0.15)	(0.13)	(0.13)	(0.15)	(0.15)	(0.13)	(0.14)	(0.13)	(0.13)	(0.14)	(0.14)

Variable		CBR.1	CBR.2	CBR.3	CBR.4	CBR.5	CBR.6	CBR.7	CBR.8	CBR.9	CBR.10	CBR.11	CBR.12
	95% CI	[0.81, 1.45]	[0.78, 1.38]	[0.70, 1.21]	[0.68, 1.20]	[0.81, 1.42]	[0.77, 1.36]	[0.72, 1.26]	[0.68, 1.22]	[0.58, 1.11]	[0.53, 1.08]	[0.61, 1.18]	[0.53, 1.09]
	β	1.06	1.04	1.02	1.01	1.05	1.03	1.03	1.02	1.02	0.99	1.01	0.98
	(SE)	(0.06)	(0.06)	(0.05)	(0.05)	(0.05)	(0.05)	(0.05)	(0.05)	(0.06)	(0.06)	(0.06)	(0.06)
Commitment $_t$	OR	1.11	0.91	0.98	0.87	1.19	0.99	1.05	0.94	**0.71**	0.74	0.77	0.78
	(SE)	(0.16)	(0.13)	(0.13)	(0.13)	(0.17)	(0.14)	(0.15)	(0.14)	**(0.12)**	(0.13)	(0.13)	(0.14)
	95% CI	[0.83, 1.48]	[0.68, 1.20]	[0.75, 1.28]	[0.65, 1.16]	[0.90, 1.56]	[0.75, 1.32]	[0.80, 1.38]	[0.70, 1.25]	**[0.51, 0.98]**	[0.52, 1.05]	[0.55, 1.08]	[0.55, 1.12]
	β	1.07	0.97	1.05	0.99	1.10	1.01	1.08	1.03	**0.95**	0.98	0.96	0.99
	(SE)	(0.06)	(0.05)	(0.05)	(0.05)	(0.06)	(0.05)	(0.05)	(0.05)	**(0.06)**	(0.06)	(0.06)	(0.06)
P&C $_t$: Commitment $_t$	OR	1.07	1.08	1.25	1.29	1.05	1.07	1.21	1.28	**1.64**	**1.66**	1.43	1.58
	(SE)	(0.22)	(0.22)	(0.24)	(0.27)	(0.21)	(0.22)	(0.24)	(0.27)	**(0.38)**	**(0.42)**	(0.34)	(0.41)
	95% CI	[0.61, 1.65]	[0.59, 1.61]	[0.85, 1.84]	[0.86, 1.94]	[0.71, 1.55]	[0.72, 2.46]	[0.82, 1.79]	[0.85, 1.93]	**[1.00, 2.60]**	**[1.01, 2.76]**	[0.85, 2.34]	[0.94, 2.64]
	β	1.02	1.02	1.06	1.07	1.01	1.02	1.05	1.06	**1.13**	**1.14**	1.10	1.12
	(SE)	(0.05)	(0.05)	(0.05)	(0.06)	(0.05)	(0.05)	(0.05)	(0.06)	**(0.07)**	**(0.07)**	(0.07)	(0.07)
SD (Intercept id)		3.22	3.62	2.70	3.11	2.86	3.36	2.68	3.10	4.56	4.38	4.52	4.44
SD (Time id)		1.01	1.01	1.02	1.02	1.01	1.01	1.02	1.02	1.01	1.01	1.01	1.01
Num.Obs.		2184	2184	2184	2184	2184	2184	2184	2184	2184	2184	2184	2184
R2 Marg.		0.050	0.062	0.046	0.074	0.048	0.066	0.058	0.083	0.048	0.057	0.050	0.052
R2 Cond.		0.328	0.376	0.265	0.335	0.287	0.355	0.273	0.340	0.440	0.433	0.438	0.434
AIC		2350.2	2454.0	2613.1	2382.5	2514.9	2475.1	2582.0	2322.5	1949.7	1680.4	1875.9	1610.6
BIC		2401.4	2505.2	2664.3	2433.7	2566.1	2526.3	2633.2	2373.7	2000.9	1731.6	1927.1	1661.8
ICC		0.3	0.3	0.2	0.3	0.3	0.3	0.2	0.3	0.4	0.4	0.4	0.4
RMSE		0.41	0.42	0.44	0.41	0.43	0.42	0.43	0.41	0.37	0.34	0.36	0.33

Note. CBR.1 is the operationalization of cue-behavior repetition as done in the main manuscript. The others reflect the labels and definitions provided in Box 1; Significant fixed effects (p-value < .05) are in bold; β = standardized effect size; Fixed effects are expressed as odds ratio from GLMER models; HS = habit strength; CBR = cue-behavior repetition; P&C = prompts and cues.

Table S11: Model 3

Model 3 (Dependent Variable is Steps) Under Different Operationalizations of Cue-Behavior Repetition

Variable		CBR.1	CBR.2	CBR.3	CBR.4	CBR.5	CBR.6	CBR.7	CBR.8	CBR.9	CBR.10	CBR.11	CBR.12
Intercept	Est.	**6290.0**	**6677.9**	**7025.3**	**7125.1**	**6863.1**	**7045.0**	**7245.7**	**7309.4**	**7624.3**	**7743.7**	**7698.9**	**7769.2**
	(SE)	**(520.0)**	**(497.7)**	**(485.8)**	**(480.3)**	**(508.3)**	**(494.5)**	**(485.1)**	**(481.7)**	**(489.4)**	**(488.2)**	**(489.5)**	**(487.9)**
	95%	**[5260.3,**	**[5692.2,**	**[6062.5,**	**[6173.1,**	**[5855.6,**	**[6065.2,**	**[6283.8,**	**[6354.0,**	**[6652.7,**	**[6773.8,**	**[6726.9,**	**[6799.6,**
	CI	**7310.5]**	**7655.2]**	**7980.0]**	**8069.1]**	**7861.3]**	**8016.4]**	**8199.3]**	**8256.4]**	**8586.3]**	**8703.8]**	**8661.2]**	**8728.6]**
	β	**-0.03**	**-0.03**	**-0.03**	**-0.03**	**-0.03**	**-0.03**	**-0.03**	**-0.03**	**-0.04**	**-0.04**	**-0.04**	**-0.04**
	(SE)	**(0.10)**	**(0.10)**	**(0.10)**	**(0.10)**	**(0.10)**	**(0.10)**	**(0.10)**	**(0.10)**	**(0.10)**	**(0.10)**	**(0.10)**	**(0.10)**
Time	Est.	2.9	3.6	1.6	2.5	1.5	2.3	1.0	1.7	-0.4	-0.6	-0.7	-0.9
	(SE)	(3.3)	(3.3)	(3.3)	(3.3)	(3.3)	(3.3)	(3.3)	(3.3)	(3.3)	(3.3)	(3.3)	(3.3)
	95%	[-3.5,	[-2.8,	[-4.8,	[-4.0,	[-5.0,	[-4.2,	[-5.5,	[-4.8,	[-6.9,	[-7.2,	[-7.2,	[-7.4,
	CI	9.3]	10.1]	8.0]	9.0]	7.9]	8.8]	7.4]	8.3]	6.1]	5.9]	5.8]	5.7]
	β	0.02	0.02	0.01	0.02	0.01	0.02	0.01	0.01	<0.01	<0.01	<0.01	-0.01
	(SE)	(0.02)	(0.02)	(0.02)	(0.02)	(0.02)	(0.02)	(0.02)	(0.02)	(0.02)	(0.02)	(0.02)	(0.02)
Steps $_{t-1}$	Est.	**0.2**	**0.2**	**0.2**	**0.2**	**0.2**	**0.2**	**0.2**	**0.2**	**0.2**	**0.2**	**0.2**	**0.2**
	(SE)	**(0.0)**	**(0.0)**	**(0.0)**	**(0.0)**	**(0.0)**	**(0.0)**	**(0.0)**	**(0.0)**	**(0.0)**	**(0.0)**	**(0.0)**	**(0.0)**
	95%	**[0.1,**	**[0.1,**	**[0.1,**	**[0.1,**	**[0.1,**	**[0.1,**	**[0.1,**	**[0.1,**	**[0.1,**	**[0.1,**	**[0.1,**	**[0.1,**
	CI	**0.2]**	**0.2]**	**0.2]**	**0.2]**	**0.2]**	**0.2]**	**0.2]**	**0.2]**	**0.2]**	**0.2]**	**0.2]**	**0.2]**
	β	**0.16**	**0.15**	**0.16**	**0.15**	**0.16**	**0.16**	**0.16**	**0.16**	**0.16**	**0.16**	**0.16**	**0.16**
	(SE)	**(0.02)**	**(0.02)**	**(0.02)**	**(0.02)**	**(0.02)**	**(0.02)**	**(0.02)**	**(0.02)**	**(0.02)**	**(0.02)**	**(0.02)**	**(0.02)**
HS $_t$	Est.	330.8	**408.1**	**447.0**	**451.2**	**432.4**	**474.1**	**492.6**	**490.5**	**637.3**	**631.6**	**650.0**	**641.5**
	(SE)	(201.8)	**(201.7)**	**(202.2)**	**(202.6)**	**(203.2)**	**(203.1)**	**(203.0)**	**(203.4)**	**(203.4)**	**(204.1)**	**(203.6)**	**(203.9)**
	95%	[-64.4,	**[13.0,**	**[50.8,**	**[54.2,**	**[34.2,**	**[76.1,**	**[94.9,**	**[91.7,**	**[238.4,**	**[231.7,**	**[251.1,**	**[241.8,**
	CI	725.5]	**802.5]**	**842.3]**	**847.2]**	**829.8]**	**871.1]**	**889.4]**	**888.1]**	**1036.3]**	**1030.6]**	**1048.0]**	**1040.2]**
	β	0.04	**0.04**	**0.05**	**0.05**	**0.05**	**0.05**	**0.05**	**0.05**	**0.07**	**0.07**	**0.07**	**0.07**
	(SE)	(0.02)	**(0.02)**	**(0.02)**	**(0.02)**	**(0.02)**	**(0.02)**	**(0.02)**	**(0.02)**	**(0.02)**	**(0.02)**	**(0.02)**	**(0.02)**
CBR $_t$	Est.	**2123.1**	**1833.0**	**1572.9**	**1582.0**	**1565.7**	**1444.5**	**1344.9**	**1360.4**	**863.3**	**734.8**	**728.5**	**710.3**
	(SE)	**(220.2)**	**(206.0)**	**(193.8)**	**(202.8)**	**(207.7)**	**(203.9)**	**(194.7)**	**(206.0)**	**(232.4)**	**(252.9)**	**(237.9)**	**(259.2)**

Variable		CBR.1	CBR.2	CBR.3	CBR.4	CBR.5	CBR.6	CBR.7	CBR.8	CBR.9	CBR.10	CBR.11	CBR.12
	95% CI	**[1691.6, 2553.4]**	**[1430.2, 2236.2]**	**[1194.2, 1953.1]**	**[1186.0, 1980.2]**	**[1159.1, 1971.9]**	**[1046.0, 1844.0]**	**[964.7, 1726.9]**	**[958.2, 1765.1]**	**[409.5, 1319.4]**	**[241.1, 1231.8]**	**[264.1, 1195.7]**	**[204.3, 1219.8]**
	β	**0.22**	**0.21**	**0.18**	**0.18**	**0.18**	**0.17**	**0.16**	**0.15**	**0.09**	**0.07**	**0.07**	**0.06**
	(SE)	**(0.02)**	**(0.02)**	**(0.02)**	**(0.02)**	**(0.02)**	**(0.02)**	**(0.02)**	**(0.02)**	**(0.02)**	**(0.02)**	**(0.02)**	**(0.02)**
$P\&C_t$	Est.	-115.3	-81.3	-29.3	-17.9	-88.8	-63.9	-29.9	-17.2	2.0	-3.4	-10.9	-4.7
	(SE)	(249.0)	(250.0)	(250.9)	(251.2)	(251.5)	(252.0)	(252.1)	(252.4)	(254.6)	(255.0)	(254.9)	(255.1)
	95% CI	[-602.7, 371.8]	[-570.7, 407.6]	[-520.4, 461.5]	[-509.8, 473.6]	[-581.3, 403.2]	[-557.2, 429.1]	[-523.5, 463.4]	[-511.4, 476.7]	[-496.5, 500.1]	[-502.6, 495.5]	[-509.9, 487.7]	[-504.0, 494.4]
	β	-0.01	-0.01	<0.01	<0.01	-0.01	<0.01	<0.01	<0.01	<0.01	<0.01	<0.01	<0.01
	(SE)	(0.02)	(0.02)	(0.02)	(0.02)	(0.02)	(0.02)	(0.02)	(0.02)	(0.02)	(0.02)	(0.02)	(0.02)
$Commitment_t$	Est.	-43.6	29.9	8.9	43.9	-76.9	-13.7	-19.2	14.8	49.6	31.9	29.0	25.0
	(SE)	(246.3)	(247.3)	(248.3)	(248.7)	(249.0)	(249.3)	(249.6)	(249.8)	(252.1)	(252.4)	(252.3)	(252.4)
	95% CI	[-525.9, 438.1]	[-454.5, 513.5]	[-477.3, 494.4]	[-443.2, 530.2]	[-564.6, 410.2]	[-502.1, 473.8]	[-508.0, 468.8]	[-474.6, 503.3]	[-444.2, 542.7]	[-462.4, 525.5]	[-465.2, 522.3]	[-469.3, 518.7]
	β	<0.01	0.01	<0.01	<0.01	<0.01	<0.01	<0.01	<0.01	<0.01	<0.01	<0.01	<0.01
	(SE)	(0.02)	(0.02)	(0.02)	(0.02)	(0.02)	(0.02)	(0.02)	(0.02)	(0.02)	(0.02)	(0.02)	(0.02)
$P\&C_t$: $Commitment_t$	Est.	63.8	46.0	-21.5	-26.1	73.8	53.1	-4.9	-16.1	-27.2	-7.3	4.8	0.9
	(SE)	(349.9)	(351.4)	(352.9)	(353.4)	(353.5)	(354.3)	(354.6)	(355.1)	(358.4)	(358.8)	(358.6)	(358.8)
	95% CI	[-620.5, 749.2]	[-641.2, 734.3]	[-711.6, 669.6]	[-717.2, 666.2]	[-617.6, 766.3]	[-639.6, 747.2]	[-698.4, 689.7]	[-710.5, 679.5]	[-728.1, 674.7]	[-709.1, 695.5]	[-696.4, 707.1]	[-700.9, 703.7]
	β	<0.01	<0.01	<0.01	<0.01	<0.01	<0.01	<0.01	<0.01	<0.01	<0.01	<0.01	<0.01
	(SE)	(0.02)	(0.02)	(0.02)	(0.02)	(0.02)	(0.02)	(0.02)	(0.02)	(0.02)	(0.02)	(0.02)	(0.02)
SD (Intercept id)		2089.5	2006.4	1977.7	1955.0	2064.0	2006.3	1985.5	1970.8	2020.9	2028.7	2026.8	2030.8
SD (Observations)		3612.7	3628.4	3642.7	3648.4	3650.0	3658.2	3661.2	3665.9	3696.2	3701.8	3700.8	3702.7
Num.Obs.		1742	1742	1742	1742	1742	1742	1742	1742	1742	1742	1742	1742
R2 Marg.		0.075	0.072	0.061	0.060	0.058	0.056	0.053	0.051	0.037	0.033	0.034	0.033
R2 Cond.		0.307	0.289	0.275	0.270	0.286	0.275	0.268	0.264	0.259	0.256	0.257	0.256
AIC		33496.8	33510.1	33523.0	33527.8	33531.6	33538.1	33540.6	33544.5	33573.6	33578.7	33577.9	33579.6
BIC		33551.4	33564.7	33577.7	33582.4	33586.2	33592.7	33595.2	33599.1	33628.2	33633.3	33632.5	33634.2

Variable	CBR.1	CBR.2	CBR.3	CBR.4	CBR.5	CBR.6	CBR.7	CBR.8	CBR.9	CBR.10	CBR.11	CBR.12
ICC	0.3	0.2	0.2	0.2	0.2	0.2	0.2	0.2	0.2	0.2	0.2	0.2
RMSE	3581.87	3597.56	3611.76	3617.44	3618.87	3627.09	3630.13	3634.77	3664.79	3670.30	3669.35	3671.24

Note. CBR.1 is the operationalization of cue-behavior repetition as done in the main manuscript. The others reflect the labels and definitions provided in Box 1; Significant fixed effects (p-value < .05) are in bold; β = standardized effect size; HS = habit strength; CBR = cue-behavior repetition; P&C = prompts and cues.

Table S12: Sensitivity Analysis After Removing Non-Wearing Days From the Coding of Cue-Behavior Repetition

Multilevel Models Summary for Sensitivity Analysis With Cue-Behavior Repetition Coded as 'Missing' Instead of 0 During Those Days When the Activity was not Recorded due to the Device not Being Worn

	Fixed effect		Random effect
**Model 1 – DV is Habit strength**	**Est. (SE)**	**95% CI**	**SD**
**Intercept**	**2.15 (0.19)**	**[1.77, 2.52]**	0.90
**Time**	**<0.01 (0.00)**	**[0.00, 0.00]**	<0.01
**HS_{t-1}**	**0.36 (0.06)**	**[0.24, 0.47]**	0.24
**CBR_t**	**0.17 (0.04)**	**[0.08, 0.25]**	0.19
$P\&C_t$	-0.05 (0.03)	[-0.12, 0.01]	-
$Commitment_t$	-0.03 (0.03)	[-0.09, 0.04]	-
$P\&C_t$:$Commitment_t$	0.06 (0.03)	[-0.00, 0.12]	-
Time:$P\&C_t$	<0.01 (0.00)	[-0.00, 0.00]	-
Time:$Commitment_t$	<0.01 (0.00)	[-0.00, 0.00]	-
Number observations = 1872; ICC = 0.9			
**Model 2 – DV is CBR**	**OR (SE)**	**95% CI**	**SD**
**Intercept**	**4.35 (1.33)**	**[2.40, 8.21]**	2.89
**Time**	**0.99 (0.00)**	**[0.98, 0.99]**	1.01
**CBR_{t-1}**	**1.35 (0.20)**	**[1.00, 1.80]**	-
HS_{t-1}	0.93 (0.14)	[0.69, 1.23]	-
$P\&C_t$	1.23 (0.21)	[0.88, 1.75]	-
$Commitment_t$	1.32 (0.23)	[0.94, 1.87]	-
$P\&C_t$:$Commitment_t$	0.88 (0.22)	[0.54, 1.43]	-
Number observations = 1797; ICC = 0.3			
**Model 3 – DV is Steps**	**Est. (SE)**	**95% CI**	**SD**
**Intercept**	**6288.23 (521.00)**	**[5256.72, 7310.47]**	2086.34
Time	2.88 (3.28)	[-3.54, 9.29]	-
**$Steps_{t-1}$**	**0.18 (0.02)**	**[0.14, 0.23]**	-
HS_t	340.66 (202.46)	[-55.77, 736.66]	-
**CBR_t**	**2107.49 (223.19)**	**[1670.07, 2543.62]**	-
$P\&C_t$	-88.56 (250.11)	[-578.16, 400.79]	-
$Commitment_t$	-4.78 (247.39)	[-489.26, 479.08]	-
$P\&C_t$:$Commitment_t$	6.63 (351.47)	[-680.74, 694.99]	-
Number observations = 1732; ICC = 0.2			

Note. Significant fixed effects are in bold; OR = odds ratio from GLMER models; DV = dependent variable; HS = habit strength; CBR = cue-behavior repetition; P&C = prompts and cues.

Table S13: Sensitivity Analysis With the Coding of Cue-Behavior Repetition Based on Garmin-Recorded Activities and Daily Evening Reports

Multilevel Models Summary for Sensitivity Analysis Where the Coding of Cue-Behavior Repetition is Based on a Triangulation of Participants' Garmin-Recorded Activities and Daily Evening Reports

	Fixed effect		Random effect
**Model 1 – DV is Habit strength**	**Est. (SE)**	**95% CI**	**SD**
**Intercept**	**2.19 (0.19)**	**[1.81, 2.56]**	0.91
**Time**	**<0.01 (0.00)**	**[0.00, 0.00]**	<0.01
**HS_{t-1}**	**0.35 (0.06)**	**[0.23, 0.46]**	0.25
**CBR_t**	**0.14 (0.04)**	**[0.06, 0.21]**	0.17
$P\&C_t$	-0.05 (0.03)	[-0.11, 0.01]	-
$Commitment_t$	-0.04 (0.03)	[-0.10, 0.02]	-
**$P\&C_t$:$Commitment_t$**	**0.06 (0.03)**	**[0.00, 0.11]**	-
Time:$P\&C_t$	<0.01 (0.00)	[-0.00, 0.00]	-
Time:$Commitment_t$	<0.01 (0.00)	[-0.00, 0.00]	-
Number observations = 2107; ICC = 0.9			
**Model 2 – DV is CBR**	**OR (SE)**	**95% CI**	**SD**
**Intercept**	**1.71 (0.46)**	**[1.00, 2.98]**	2.90
**Time**	**0.99 (0.00)**	**[0.99, 1.00]**	1.01
**CBR_{t-1}**	**1.40 (0.16)**	**[1.12, 1.74]**	-
HS_{t-1}	1.20 (0.15)	[0.93, 1.53]	-
**$P\&C_t$**	**1.49 (0.21)**	**[1.13, 1.98]**	-
$Commitment_t$	1.28 (0.18)	[0.97, 1.69]	-
$P\&C_t$:$Commitment_t$	0.81 (0.16)	[0.54, 1.21]	-
Number observations = 2184; ICC = 0.3			
**Model 3 – DV is Steps**	**Est. (SE)**	**95% CI**	**SD**
**Intercept**	**7189.44 (510.75)**	**[6175.93, 8192.09]**	2080.48
Time	-0.07 (3.30)	[-6.54, 6.39]	-
**$Steps_{t-1}$**	**0.18 (0.02)**	**[0.13, 0.23]**	-
**HS_t**	**478.89 (205.54)**	**[76.34, 880.86]**	-
**CBR_t**	**1170.33 (215.83)**	**[747.63, 1592.27]**	-
$P\&C_t$	-132.49 (254.13)	[-629.95, 364.975	-
$Commitment_t$	-58.62 (251.01)	[-550.23, 432.31]	-
$P\&C_t$:$Commitment_t$	94.01 (356.39)	[-603.00, 792.01]	-
Number observations = 1742; ICC = 0.2			

Note. Cue-behavior repetition was coded as 1 under the following conditions: a) Both a Garmin-recorded activity and a brisk walk reported in the evening questionnaire were present; b) A Garmin-recorded activity was present, but the participant did not complete the evening questionnaire; c) No Garmin-recorded activity

was present, but the brisk walk was reported in the evening questionnaire. Cue-behavior repetition was coded as 0 in any other instance. Significant fixed effects are in bold; OR = odds ratio from GLMER models; DV = dependent variable; HS = habit strength; CBR = cue-behavior repetition; P&C = prompts and cues.

Table S14: Sensitivity Analysis Including all the Participants who Entered the Intervention Phase

Multilevel Models Summary Including all the Participants who Entered the Intervention Phase

	Fixed effect		Random effect
**Model 1 – DV is Habit strength**	**Est. (SE)**	**95% CI**	**SD**
**Intercept**	**2.00 (0.14)**	**[1.72, 2.29]**	0.86
Time	<0.01 (0.00)	[0.00, 0.00]	<0.01
**HS_{t-1}**	**0.36 (0.05)**	**[0.26, 0.46]**	0.24
**CBR_t**	**0.14 (0.03)**	**[0.08, 0.21]**	0.16
**$P\&C_t$**	**-0.07 (0.03)**	**[-0.12, -0.01]**	-
$Commitment_t$	-0.03 (0.03)	[-0.09, 0.02]	-
**$P\&C_t$:$Commitment_t$**	**0.06 (0.03)**	**[0.01, 0.11]**	-
Time:$P\&C_t$	<0.01 (0.00)	[-0.00, 0.00]	-
Time:$Commitment_t$	<0.01 (0.00)	[-0.00, 0.00]	-
Number observations = 2385; ICC = 0.9			
**Model 2 – DV is CBR**	**OR (SE)**	**95% CI**	**SD**
**Intercept**	**1.84 (0.52)**	**[1.06, 3.21]**	4.14
**Time**	**0.98 (0.00)**	**[0.97, 0.99]**	1.01
**CBR_{t-1}**	**1.45 (0.16)**	**[1.17, 1.81]**	-
HS_{t-1}	1.18 (0.15)	[0.92, 1.50]	-
$P\&C_t$	1.07 (0.15)	[0.82, 1.40]	-
$Commitment_t$	1.00 (0.14)	[0.77, 1.32]	-
$P\&C_t$:$Commitment_t$	1.10 (0.22)	[0.75, 1.61]	-
Number observations = 2539; ICC = 0.4			
**Model 3 – DV is Steps**	**Est. (SE)**	**95% CI**	**SD**
**Intercept**	**6184.57 (439.28)**	**[5317.56, 7044.96]**	2111.06
Time	4.36 (3.14)	[-1.79, 10.51]	-
**$Steps_{t-1}$**	**0.17 (0.02)**	**[0.13, 0.22]**	-
HS_t	318.43 (196.27)	[-66.46, 702.12]	-
**CBR_t**	**2030.81 (206.96)**	**[1624.93, 2435.29]**	-
$P\&C_t$	-235.43 (237.44)	[-700.26, 229.21]	-
$Commitment_t$	-113.96 (235.90)	[-575.94, 347.54]	-
$P\&C_t$:$Commitment_t$	147.38 (334.24)	[-506.76, 801.65]	-
Number observations = 1933; ICC = 0.3			

Note. Significant fixed effects are in bold; OR = odds ratio from GLMER models; DV = dependent variable; HS = habit strength; CBR = cue-behavior repetition; P&C = prompts and cues.

Table S15: N-of-1 Level Results for Model 1

N-of-1 Level Results for Model 1 (Dependent Variable is Habit Strength) With the Target Operationalization of Cue-Behavior Repetition (CBR.1) for Participants with Less Than 20% Missing Values during the First 66 days of the Habit Strength Time Series

PART A

Variable		P1 (GAM)	P11 (constant)	P5 (constant)	P24 (linear)	P6 (linear)	P21 (GAM)	P18 (asymptotic)*	P13 (constant)
Intercept	Est. (SE)	**1.60 (0.07)**	**3.74 (0.11)**	**0.98 (0.06)**	**3.59 (0.18)**	**2.60 (0.13)**	**2.62 (0.06)**	**2.84 (0.07)**	**1.27 (0.03)**
	95% CI	**[1.47, 1.73]**	**[3.52, 3.95]**	**[0.87, 1.10]**	**[3.24, 3.94]**	**[2.33, 2.86]**	**[2.51, 2.74]**	**[2.70, 2.97]**	**[1.21, 1.32]**
HS_{t-1}	Est. (SE)	**0.61 (0.07)**	**0.26 (0.10)**	-0.02 (0.09)	-0.01 (0.11)	**-0.20 (0.10)**	**0.24 (0.09)**	0.16 (0.10)	**0.41 (0.09)**
	95% CI	**[0.47, 0.74]**	**[0.07, 0.45]**	[-0.20, 0.16]	[-0.22, 0.20]	**[-0.39, 0.00]**	**[0.07, 0.42]**	[-0.03, 0.35]	**[0.22, 0.59]**
CBR_{t}	Est. (SE)	**0.19 (0.07)**	**0.25 (0.10)**	**0.31 (0.05)**	**0.24 (0.10)**	-0.01 (0.09)	**0.17 (0.06)**	**0.23 (0.06)**	-0.02 (0.03)
	95% CI	**[0.05, 0.32]**	**[0.04, 0.46]**	**[0.21, 0.41]**	**[0.04, 0.44]**	[-0.19, 0.17]	**[0.07, 0.28]**	**[0.11, 0.36]**	[-0.07, 0.03]
$P\&C_{t}$	Est. (SE)	-0.01 (0.06)	0.01 (0.18)	**-0.26 (0.09)**	-0.10 (0.17)	-0.01 (0.12)	0.09 (0.07)	0.02 (0.14)	0.07 (0.05)
	95% CI	[-0.14, 0.12]	[-0.34, 0.37]	**[-0.45, -0.08]**	[-0.45, 0.24]	[-0.25, 0.23]	[-0.06, 0.24]	[-0.27, 0.31]	[-0.03, 0.16]
$Commitment_{t}$	Est. (SE)	**0.16 (0.06)**	-0.08 (0.18)	-0.02 (0.09)	0.12 (0.18)	**-0.26 (0.12)**	-0.05 (0.08)	-0.17 (0.15)	0.09 (0.05)
	95% CI	**[0.04, 0.29]**	[-0.44, 0.27]	[-0.20, 0.16]	[-0.23, 0.47]	**[-0.50, -0.01]**	[-0.20, 0.10]	[-0.46, 0.13]	[-0.01, 0.18]
$P\&C_{t}$: $Commitment_{t}$	Est. (SE)	0.03 (0.06)	0.23 (0.17)	0.00 (0.09)	0.01 (0.15)	0.06 (0.11)	0.00 (0.07)	**0.26 (0.12)**	-0.07 (0.05)
	95% CI	[-0.08, 0.14]	[-0.10, 0.57]	[-0.17, 0.18]	[-0.30, 0.31]	[-0.16, 0.27]	[-0.13, 0.13]	**[0.01, 0.51]**	[-0.16, 0.03]

Variable		P1 (GAM)	P11 (constant)	P5 (constant)	P24 (linear)	P6 (linear)	P21 (GAM)	P18 (asymptotic)*	P13 (constant)
Time: P&C $_t$	Est. (SE)	0.00 (0.00)	0.00 (0.00)	**0.00 (0.00)**	0.00 (0.00)	0.00 (0.00)	0.00 (0.00)	0.00 (0.00)	0.00 (0.00)
	95% CI	[0.00, 0.00]	[0.00, 0.00]	**[0.00, 0.01]**	[0.00, 0.01]	[0.00, 0.00]	[0.00, 0.00]	[-0.01, 0.00]	[0.00, 0.00]
Time: Commitment $_t$	Est. (SE)	**0.00 (0.00)**	0.00 (0.00)	0.00 (0.00)	0.00 (0.00)	0.00 (0.00)	0.00 (0.00)	0.00 (0.00)	0.00 (0.00)
	95% CI	**[0.00, 0.00]**	[0.00, 0.00]	[0.00, 0.00]	[-0.01, 0.00]	[0.00, 0.01]	[0.00, 0.00]	[0.00, 0.00]	[0.00, 0.00]
Num.Obs.		104	104	104	104	104	104	104	97
R2		0.894	0.131	0.315	0.273	0.227	0.919	0.136	0.260
R2 Adj.		-	0.068	0.265	0.212	0.162	-	-	0.202
AIC		-108.5	122.9	-3.7	105.9	32.7	-63.0	66.1	-132.7
BIC		-82.0	146.7	20.1	132.3	59.2	-18.4	92.6	-109.5
Log.Lik.		-	-52.468	10.828	-42.951	-6.365	-	-	75.336
RMSE		0.13	0.40	0.22	0.37	0.26	0.15	0.30	0.11

PART B

Variable		P15 (quadratic)	P2 (GAM)	P3 (GAM)	P8 (GAM)	P12 (linear)	P9 (linear)	P16 (quadratic)	P23 (GAM)
Intercept	Est. (SE)	**2.05 (0.28)**	**2.29 (0.06)**	**1.73 (0.09)**	**3.66 (0.03)**	**0.88 (0.15)**	**1.15 (0.11)**	**1.54 (0.43)**	**1.62 (0.06)**
	95% CI	**[1.49, 2.61]**	**[2.18, 2.41]**	**[1.54, 1.91]**	**[3.61, 371]**	**[0.59, 1.17]**	**[0.92, 1.37]**	**[0.67, 2.40]**	**[1.50, 1.75]**
HS $_{t-1}$	Est. (SE)	0.04 (0.11)	0.18 (0.10)	**0.40 (0.07)**	0.13 (0.10)	0.05 (0.12)	**0.28 (0.10)**	0.10 (0.13)	**0.63 (0.10)**
	95% CI	[-0.17, 0.26]	[-0.02, 0.38]	**[0.27, 0.53]**	[-0.06, 0.32]	[-0.19, 0.29]	**[0.08, 0.48]**	[-0.16, 0.36]	**[0.42, 0.84]**
CBR $_t$	Est. (SE)	-0.12 (0.18)	0.11 (0.06)	**0.81 (0.10)**	-0.01 (0.02)	0.08 (0.08)	**0.24 (0.06)**	**0.44 (0.20)**	0.13 (0.08)

Variable		P15 (quadratic)	P2 (GAM)	P3 (GAM)	P8 (GAM)	P12 (linear)	P9 (linear)	P16 (quadratic)	P23 (GAM)
	95% CI	[-0.48, 0.25]	[-0.01, 0.24]	**[0.61, 1.01]**	[-0.06, 0.04]	[-0.08, 0.25]	**[0.12, 0.36]**	**[0.03, 0.84]**	[-0.02, 0.28]
P&C $_t$	Est. (SE)	-0.14 (0.24)	0.09 (0.12)	-0.25 (0.20)	-0.02 (0.04)	-0.09 (0.13)	0.11 (0.13)	-0.51 (0.42)	0.06 (0.15)
	95% CI	[-0.61, 0.34]	[-0.15, 0.33]	[-0.65, 0.14]	[-0.11, 0.07]	[-0.35, 0.16]	[-0.16, 0.38]	[-1.34, 0.32]	[-0.24, 0.36]
Commitment $_t$	Est. (SE)	-0.13 (0.24)	0.02 (0.12)	-0.36 (0.20)	0.01 (0.04)	-0.02 (0.13)	0.02 (0.14)	-0.67 (0.43)	**0.31 (0.14)**
	95% CI	[-0.60, 0.34]	[-0.22, 0.25]	[-0.76, 0.04]	[-0.07, 0.10]	[-0.29, 0.24]	[-0.25, 0.30]	[-1.52, 0.18]	**[0.02, 0.59]**
P&C $_t$: Commitment $_t$	Est. (SE)	-0.07 (0.22)	-0.02 (0.11)	**0.37 (0.18)**	0.04 (0.04)	0.06 (0.11)	-0.07 (0.12)	0.02 (0.35)	0.02 (0.13)
	95% CI	[-0.50, 0.36]	[-0.24, 0.20]	**[0.01, 0.73]**	[-0.04, 0.12]	[-0.16, 0.27]	[-0.31, 0.17]	[-0.68, 0.72]	[-0.24, 0.29]
Time: P&C $_t$	Est. (SE)	0.00 (0.00)	0.00 (0.00)	0.00 (0.00)	0.00 (0.00)	0.00 (0.00)	0.00 (0.00)	0.01 (0.01)	0.00 (0.00)
	95% CI	[0.00, 0.01]	[0.00, 0.00]	[-0.01, 0.01]	[0.00, 0.00]	[0.00, 0.01]	[-0.01, 0.00]	[-0.01, 0.03]	[-0.01, 0.01]
Time: Commitment $_t$	Est. (SE)	0.00 (0.00)	0.00 (0.00)	0.00 (0.00)	0.00 (0.00)	0.00 (0.00)	0.00 (0.00)	0.02 (0.01)	**-0.01 (0.00)**
	95% CI	[0.00, 0.01]	[0.00, 0.00]	[-0.01, 0.01]	[0.00, 0.00]	[-0.01, 0.00]	[0.00, 0.00]	[0.00, 0.04]	**[-0.01, 0.00]**
Num.Obs.		100	102	100	97	80	83	67	59
R2		0.317	0.734	0.641	0.586	0.832	0.362	0.368	0.787
R2 Adj.		0.249	-	-	-	0.813	0.293	0.269	-
AIC		167.3	32.6	132.0	-169.6	2.0	25.0	153.4	6.6
BIC		195.9	77.3	164.1	-134.8	25.8	49.2	177.6	32.1
Log.Lik.		-72.635	-	-	-	8.994	-2.499	-65.678	-
RMSE		0.50	0.24	0.41	0.09	0.22	0.25	0.64	0.21

Variable	P15 (quadratic)	P2 (GAM)	P3 (GAM)	P8 (GAM)	P12 (linear)	P9 (linear)	P16 (quadratic)	P23 (GAM)

Note. Participants are sorted in ascending order based on the number of missing values in the habit strength time series; Significant fixed effects (*p*-value < .05) are in bold; HS = habit strength; CBR = cue-behavior repetition; P&C = prompts and cues. The models are based on the best fitting model for habit strength. The variable time was included in all models but is removed in this table for better readability. *For calculation the asymptotic model was replaced by the GAM model. Some participants exhibited very high occurrences of cue-behavior repetition (see Table S8), leading to limited variance in this variable and, ultimately, less accurate estimates of the effects.

Figure S5: N-of-1 Level Results for Model 1 - FOREST PLOT

N-of-1 Level Results for Model 1 (Dependent Variable is Habit Strength) With the Target Operationalization of Cue-Behavior Repetition (CBR.1) for Participants with Less Than 20% Missing Values during the First 66 days of the Habit Strength Time Series

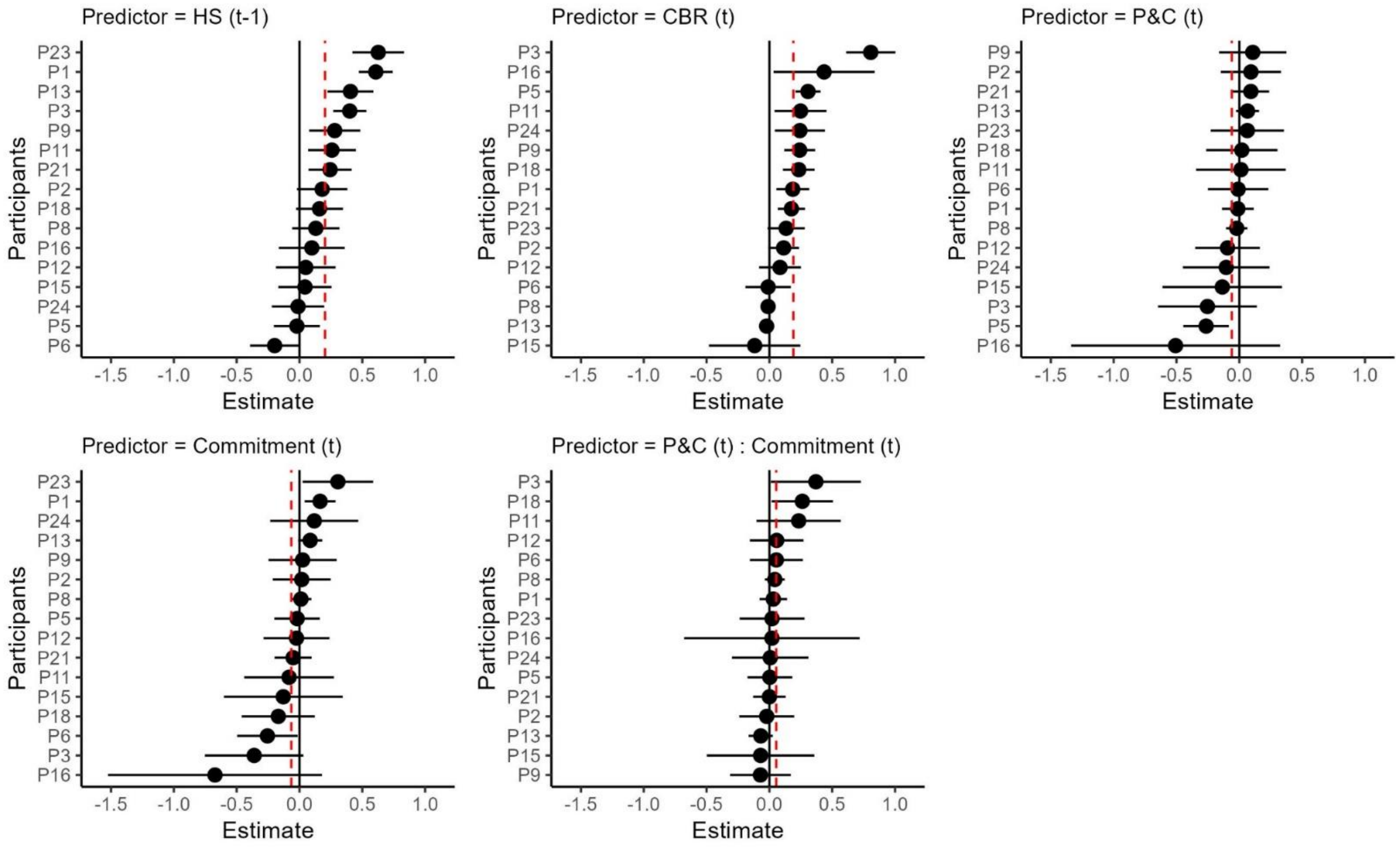

Note. The dashed red line represents the average estimate across participants. HS = habit strength; CBR = cue-behavior repetition; P&C = prompts and cues; (t) = the predictor measurement is taken on the same day as the dependent variable; (t-1) = the predictor measurement is taken on the previous day relative to the dependent variable. Some participants exhibited very high occurrences of cue-behavior repetition (see Table S8), leading to limited variance in this variable and, ultimately, less accurate estimates of the effects.

Table S16: N-of-1 Level Results for Model 2

N-of-1 Level Results for Model 2 (Dependent Variable is Cue-Behavior Repetition) With the Target Operationalization of Cue-Behavior Repetition (CBR.1) for Participants with Less Than 20% Missing Values during the First 66 days of the Habit Strength Time Series

PART A

Variable		P1	P11	P5	P24	P6	P21	P18	P13
Intercept	Est. (SE)	140279823.10 (1.49 x 10^{12})	**16.51 (15.94)**	**6.18 (5.17)**	2.49 (2.42)	211529375.96 (4.30 x 10^{11})	2.58 (4.91)	0.62 (0.39)	1.76 (1.21)
	95% CI	[0.00, -]	**[2.89, 133.22]**	**[1.26, 34.50]**	[0.39, 18.45]	[0.00, -]	[0.07, 134.28]	[0.18, 2.14]	[0.45, 6.93]
Time	Est. (SE)	1.05 (0.03)	0.98 (0.01)	**0.97 (0.01)**	1.00 (0.01)	1.00 (0.01)	1.01 (0.03)	1.00 (0.01)	**0.97 (0.01)**
	95% CI	[1.00, 1.13]	[0.96, 1.00]	**[0.95, 0.99]**	[0.98, 1.02]	[0.98, 1.03]	[0.95, 1.07]	[0.98, 1.01]	**[0.95, 0.99]**
CBR $_{t-1}$	Est. (SE)	0.00 (0.00)	0.53 (0.38)	3.22 (1.96)	1.55 (1.05)	0.00 (0.00)	2.29 (1.93)	2.30 (1.06)	1.44 (0.72)
	95% CI	[-,-]	[0.11, 1.97]	[1.00, 11.23]	[0.37, 5.68]	[-, 1.41 x 10^{44}]	[0.39, 11.57]	[0.95, 5.85]	[0.53, 3.85]
HS $_{t-1}$	Est. (SE)	0.01 (0.03)	0.44 (0.28)	2.19 (2.23)	0.64 (0.48)	2.44 (3.16)	0.21 (0.33)	**0.20 (0.15)**	2.14 (3.52)
	95% CI	[0.00, 0.86]	[0.12, 1.50]	[0.30, 20.77]	[0.14, 2.70]	[0.24, 41.03]	[0.01, 4.14]	**[0.04, 0.78]**	[0.08, 75.77]
P&C $_t$	Est. (SE)	297033178.34 (1.61 x 10^{12})	1.42 (1.04)	1.10 (0.73)	0.69 (0.47)	0.37 (45.00)	1.69 (1.70)	1.97 (1.17)	1.31 (0.86)
	95% CI	[0.00, -]	[0.35, 6.46]	[0.30, 4.11]	[0.17, 2.61]	[0.02, 3.21]	[0.23, 14.69]	[0.62, 6.51]	[0.36, 4.92]
Commitment $_t$	Est. (SE)	2.25 (2.46)	3.82 (3.43)	**0.11 (0.08)**	1.02 (0.73)	0.39 (0.47)	0.58 (0.47)	1.23 (0.72)	1.38 (0.92)

Variable		P1	P11	P5	P24	P6	P21	P18	P13
	95% CI	[0.28, 26.45]	[0.76, 29.46]	**[0.02, 0.45]**	[0.24, 4.30]	[0.02, 3.35]	[0.10, 2.72]	[0.39, 3.92]	[0.37, 5.30]
P&C $_t$: Commitment $_t$	Est. (SE)	0.92 (6926.56)	0.17 (0.19)	1.80 (1.68)	4.04 (4.55)	2.10 (3.14)	3.55 (5.39)	0.89 (0.74)	0.35 (0.34)
	95% CI	[0.00, 3.94 x 10^{162}]	[0.01, 1.43]	[0.29, 11.58]	[0.48, 44.11]	[0.13, 64.29]	[0.20, 112.86]	[0.17, 4.58]	[0.05, 2.22]
Num.Obs.		104	104	104	104	104	104	104	100
AIC		41.4	109.7	124.9	108.9	76.0	77.8	147.5	126.3
BIC		59.9	128.2	143.5	127.5	94.5	96.3	166.0	144.5
Log.Lik.		-13.705	-47.836	-55.474	-47.473	-30.996	-31.915	-66.727	-56.128
RMSE		0.19	0.38	0.42	0.38	0.29	0.30	0.47	0.43

PART B

Variable		P15	P2	P3	P8	P12	P9	P16	P23
Intercept	Est. (SE)	2.09 (3.20)	0.76 (0.60)	0.39 (0.25)	**15.71 (16.01)**	30.84 (63.32)	0.54 (0.41)	3.07 (2.75)	9.09 (11.64)
	95% CI	[0.10, 47.26]	[0.16, 3.56]	[0.11, 1.32]	**[2.40, 136.57]**	[0.78, 3100.17]	[0.12, 2.39]	[0.57, 19.96]	[0.83, 138.86]
Time	Est. (SE)	0.99 (0.01)	0.98 (0.01)	1.00 (0.01)	**0.97 (0.01)**	0.97 (0.03)	0.99 (0.01)	0.98 (0.01)	**0.91 (0.03)**
	95% CI	[0.96, 1.01]	[0.96, 1.00]	[0.98, 1.01]	**[0.95, 1.00]**	[0.90, 1.04]	[0.97, 1.01]	[0.95, 1.01]	**[0.85, 0.96]**
CBR $_{t-1}$	Est. (SE)	6.39 (6.70)	1.98 (0.98)	2.12 (1.17)	0.66 (0.40)	0.48 (0.57)	2.15 (1.14)	0.86 (0.54)	0.94 (0.69)
	95% CI	[0.88, 62.79]	[0.75, 5.32]	[0.71, 6.41]	[0.18, 2.04]	[0.02, 3.50]	[0.77, 6.20]	[0.24, 2.83]	[0.21, 3.92]
HS $_{t-1}$	Est. (SE)	0.42 (0.27)	1.61 (0.96)	1.67 (0.61)	0.06 (0.14)	0.92 (1.43)	1.35 (1.25)	1.55 (0.61)	0.38 (0.38)
	95% CI	[0.11, 1.44]	[0.50, 5.29]	[0.81, 3.48]	[0.00, 5.12]	[0.05, 23.61]	[0.22, 8.96]	[0.75, 3.53]	[0.05, 2.35]

Variable		P15	P2	P3	P8	P12	P9	P16	P23
P&C $_t$	Est. (SE)	1.21 (1.06)	1.72 (1.08)	1.22 (0.77)	1.49 (1.01)	4.85 (5.84)	**5.31 (3.71)**	1.93 (1.43)	0.50 (0.54)
	95% CI	[0.22, 7.42]	[0.51, 6.08]	[0.35, 4.32]	[0.40, 5.88]	[0.59, 104.23]	**[1.42, 22.52]**	[0.45, 8.75]	[0.05, 3.87]
Commitment $_t$	Est. (SE)	**13.17 (17.47)**	1.68 (1.07)	1.10 (0.71)	1.97 (1.45)	1.36 (1.21)	3.34 (2.29)	1.01 (0.74)	1.87 (1.79)
	95% CI	**[1.43, 354.15]**	[0.49, 6.07]	[0.30, 3.97]	[0.48, 9.02]	[0.24, 8.54]	[0.90, 13.53]	[0.24, 4.29]	[0.28, 12.97]
P&C $_t$: Commitment $_t$	Est. (SE)	0.19 (0.33)	3.25 (3.01)	0.47 (0.44)	0.30 (0.30)	0.28 (0.43)	**0.10 (0.09)**	1.73 (1.95)	2.37 (3.53)
	95% CI	[0.00, 4.58]	[0.54, 20.80]	[0.07, 2.89]	[0.04, 2.01]	[0.01, 5.01]	**[0.01, 0.60]**	[0.19, 16.69]	[0.13, 49.49]
Num.Obs.		102	103	102	101	81	86	68	62
AIC		72.0	132.0	133.2	119.4	72.0	119.2	94.1	74.4
BIC		90.4	150.4	151.6	137.7	88.7	136.4	109.6	89.3
Log.Lik.		-29.019	-58.989	-59.622	-52.710	-28.983	-52.596	-40.044	-30.183
RMSE		0.29	0.44	0.45	0.42	0.33	0.46	0.45	0.40

Note. Participants are sorted in ascending order based on the number of missing values in the habit strength time series; Significant fixed effects (p-value < .05) are in bold; High values result from low variance in CBR; HS = habit strength; CBR = cue-behavior repetition; P&C = prompts and cues. Some participants exhibited very high occurrences of cue-behavior repetition (see Table S8), leading to limited variance in this variable and, ultimately, less accurate estimates of the effects.

Figure S6: N-of-1 Level Results for Model 2 – FOREST PLOT

N-of-1 Level Results for Model 2 (Dependent Variable is Cue-Behavior Repetition) With the Target Operationalization of Cue-Behavior Repetition (CBR.1) for Participants with Less Than 20% Missing Values in the Habit Strength Time Series

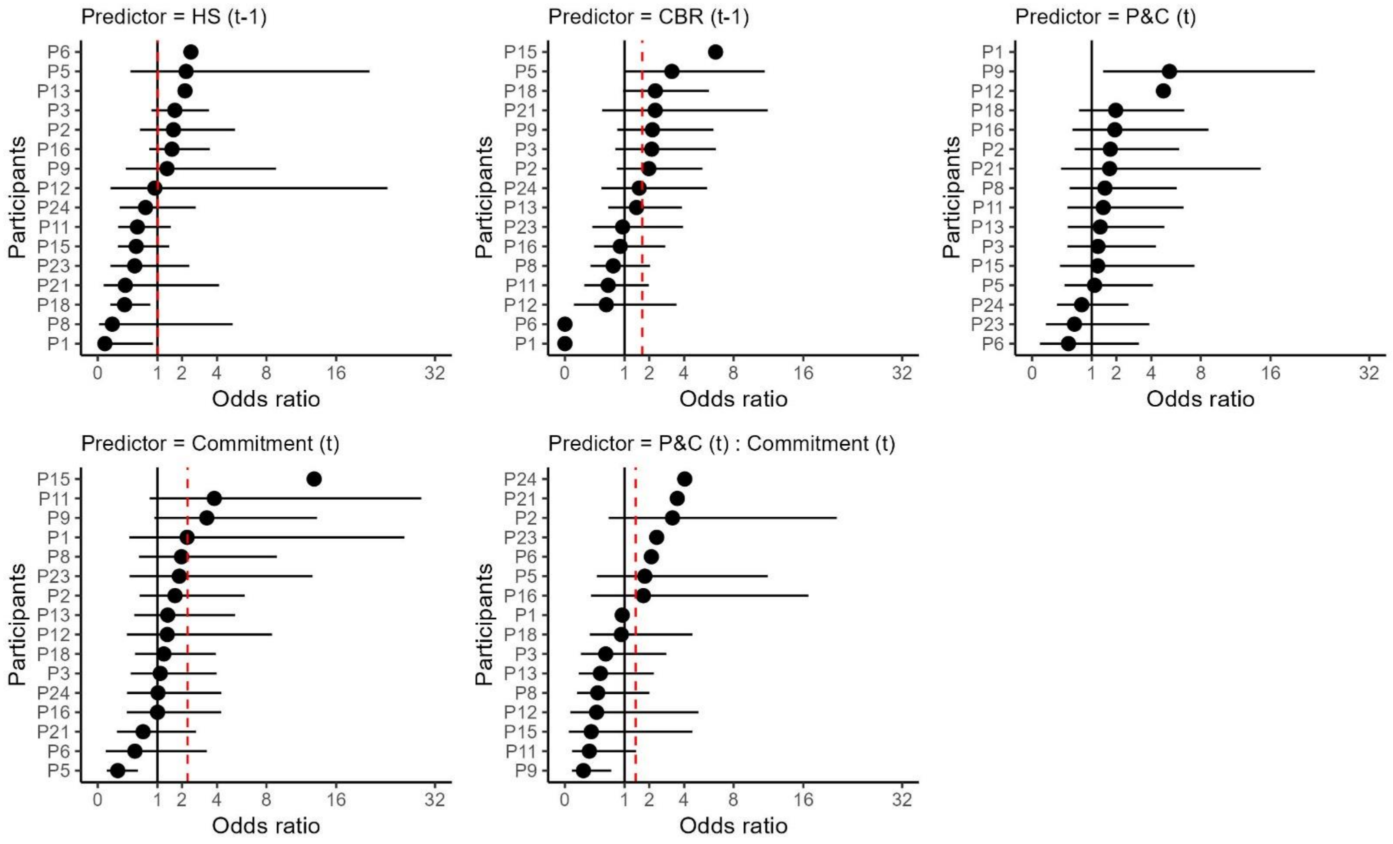

Note. The x-axis is square rooted to its values. The dashed red line represents the average odds ratio across participants. The average odds ratio for the predictor P&C falls outside the x-axis range due to a high estimate from one participant (P1). HS = habit strength; CBR = cue-behavior repetition; P&C = prompts and cues; (t) = the predictor measurement is taken on the same day as the dependent variable; (t-1) = the predictor measurement is taken on the previous day relative to the dependent variable. Estimates or confidence intervals that fall outside the range shown can be interpreted as non-significant.

Table S17: N-of-1 Level Results for Model 3

N-of-1 Level Results for Model 3 (Dependent Variable is Steps) With the Target Operationalization of Cue-Behavior Repetition (CBR.1) for Participants with Less Than 20% Missing Values in the Habit Strength Time Series

PART A

Variable		P1	P11	P5	P24	P6	P21	P18	P13
Intercept	Est.	**6778.35**	**12461.56**	1540.78	**4394.78**	1801.41	**11252.54**	**4833.25**	**6929.08**
	(SE)	**(2495.42)**	**(2113.14)**	(3014.54)	**(1256.78)**	(1647.68)	**(2408.54)**	**(693.74)**	**(1160.88)**
	95%	**[1822.23,**	**[8250.08,**	[-4556.70,	**[1893.22,**	[-1481.68,	**[6464.52,**	**[3455.01,**	**[4621.72,**
	CI	**11734.48]**	**16673.05]**	7638.25]	**6896.34]**	5084.49]	**16040.55]**	**6211.49]**	**9236.45]**
Time	Est.	17.88	-30.30	30.81	20.42	22.13	-4.90	5.66	8.85
	(SE)	(31.89)	(20.60)	(30.20)	(12.97)	(13.03)	(35.50)	(8.11)	(13.54)
	95%	[-45.46,	[-71.35,	[-30.28,	[-5.40,	[-3.83,	[-75.47,	[-10.44,	[-18.07,
	CI	81.21]	10.76]	91.90]	46.24]	48.09]	65.66]	21.77]	35.76]
Steps $_{t-1}$	Est.	0.15	**0.32**	0.18	-0.16	-0.02	**0.23**	0.16	**0.24**
	(SE)	(0.11)	**(0.11)**	(0.16)	(0.11)	(0.10)	**(0.10)**	(0.10)	**(0.10)**
	95%	[-0.06,	**[0.10,**	[-0.15,	[-0.37,	[-0.23,	**[0.02,**	[-0.03,	**[0.04,**
	CI	0.36]	**0.54]**	0.50]	0.06]	0.18]	**0.44]**	0.35]	**0.45]**
HS $_t$	Est.	-778.16	841.39	-954.75	1725.40	192.80	-120.81	1311.57	-4330.09
	(SE)	(2266.29)	(1383.08)	(2641.23)	(988.18)	(1433.80)	(1885.05)	(742.27)	(3314.53)
	95%	[-5279.20,	[-1915.08,	[-6297.14,	[-241.54,	[-2664.11,	[-3868.18,	[-163.07,	[-10918.08,
	CI	3722.87]	3597.87]	4387.64]	3692.33]	3049.71]	3626.55]	2786.21]	2257.90]
CBR $_t$	Est.	**4883.50**	393.73	**4850.03**	**2418.58**	2507.16	-1288.41	**1974.13**	1413.27
	(SE)	**(1726.69)**	(1700.09)	**(2222.72)**	**(852.90)**	(1447.02)	(1285.25)	**(520.73)**	(832.61)
	95%	**[1454.15,**	[-2994.54,	**[354.15,**	**[720.93,**	[-376.08,	[-3843.40,	**[939.60,**	[-241.65,
	CI	**8312.85]**	3782.00]	**9345.90]**	**4116.23]**	5390.41]	1266.59]	**3008.66]**	3068.18]
P&C $_t$	Est.	-1423.67	2583.61	580.88	-158.35	**3486.09**	1298.81	407.27	347.38
	(SE)	(1080.40)	(1735.46)	(1898.78)	(868.61)	**(890.71)**	(1161.35)	(683.75)	(1061.30)
	95%	[-3569.44,	[-875.15,	[-3259.77,	[-1887.28,	**[1711.30,**	[-1009.87,	[-951.12,	[-1762.06,
	CI	722.10]	6042.37]	4421.54]	1570.58]	**5260.87]**	3607.50]	1765.66]	2456.83]

Variable		P1	P11	P5	P24	P6	P21	P18	P13
Commitment $_t$	Est.	-353.98	1556.09	1072.34	-394.69	1779.08	1691.80	-499.13	1296.48
	(SE)	(1063.34)	(1636.66)	(2044.69)	(872.04)	(902.60)	(1169.77)	(678.26)	(1057.40)
	95%	[-2465.87,	[-1705.77,	[-3063.44,	[-2130.44,	[-19.38,	[-633.63,	[-1846.61,	[-805.22,
	CI	1757.90]	4817.96]	5208.11]	1341.07]	3577.54]	4017.23]	848.34]	3398.18]
P&C $_t$:	Est.	474.02	**-5511.62**	-151.97	-147.86	**-4043.70**	-1202.35	-192.74	723.44
Commitment $_t$	(SE)	(1516.59)	**(2481.86)**	(2788.10)	(1248.62)	**(1268.50)**	(1638.22)	(959.10)	(1479.37)
	95%	[-2538.06,	**[-10457.95,**	[-5791.44,	[-2633.17,	**[-6571.25,**	[-4459.03,	[-2098.15,	[-2216.98,
	CI	3486.10]	**-565.29]**	5487.50]	2337.44]	**-1516.15]**	2054.33]	1712.67]	3663.86]
Num.Obs.		100	81	47	87	82	94	98	95
R2		0.110	0.178	0.157	0.233	0.249	0.101	0.249	0.179
R2 Adj.		0.042	0.099	0.006	0.165	0.178	0.028	0.191	0.113
AIC		1933.7	1621.3	937.8	1640.7	1543.9	1830.8	1806.2	1830.8
BIC		1957.1	1642.9	954.4	1662.9	1565.6	1853.7	1829.4	1853.8
Log.Lik.		-957.829	-801.674	-459.890	-811.342	-762.952	-906.381	-894.078	-906.403
RMSE		3495.91	4809.14	4298.21	2715.76	2658.07	3727.22	2217.89	3368.23

PART B

Variable		P15	P2	P3	P8	P12	P9	P16	P23
Intercept	Est.	2910.82	**6741.55**	**8245.25**	**8801.83**	**8468.97**	**5039.12**	**3817.29**	**8355.85**
	(SE)	(1707.66)	**(1262.54)**	**(968.06)**	**(1267.31)**	**(1406.64)**	**(1121.65)**	**(1881.62)**	**(2647.70)**
	95%	[-487.53,	**[4228.03,**	**[6320.82,**	**[6282.92,**	**[5664.21,**	**[2777.11,**	**[37.95,**	**[3012.57,**
	CI	6309.18]	**9255.08]**	**10169.69]**	**11320.74]**	**11273.74]**	**7301.14]**	**7596.63]**	**13699.13]**
Time	Est.	7.32	-13.03	5.84	**-39.55**	-0.59	0.60	**72.53**	31.38
	(SE)	(13.44)	(16.62)	(12.77)	**(16.25)**	(27.34)	(15.88)	**(32.90)**	(61.42)
	95%	[-19.42,	[-46.12,	[-19.54,	**[-71.85,**	[-55.11,	[-31.43,	**[6.45,**	[-92.57,
	CI	34.06]	20.05]	31.23]	**-7.26]**	53.93]	32.63]	**138.61]**	155.33]
Steps $_{t-1}$	Est.	**0.27**	0.14	**0.30**	0.14	-0.08	**0.60**	0.07	**0.41**
	(SE)	**(0.11)**	(0.10)	**(0.10)**	(0.10)	(0.12)	**(0.13)**	(0.14)	**(0.15)**
	95%	**[0.05,**	[-0.06,	**[0.10,**	[-0.07,	[-0.32,	**[0.33,**	[-0.21,	**[0.11,**
	CI	**0.50]**	0.33]	**0.50]**	0.35]	0.16]	**0.87]**	0.35]	**0.71]**

Variable		P15	P2	P3	P8	P12	P9	P16	P23
HS $_t$	Est.	-50.13	-1736.81	489.08	1938.45	-1277.15	644.15	284.95	1196.44
	(SE)	(599.50)	(888.19)	(616.71)	(3120.01)	(1151.67)	(1390.19)	(727.26)	(2051.55)
	95%	[-1243.17,	[-3505.05,	[-736.89,	[-4262.90,	[-3573.52,	[-2159.42,	[-1175.79,	[-2943.76,
	CI	1142.91]	31.43]	1715.05]	8139.80]	1019.22]	3447.73]	1745.70]	5336.63]
CBR $_t$	Est.	1763.69	**3856.86**	1912.25	599.95	1417.68	753.38	1721.38	2955.76
	(SE)	(1459.33)	**(732.55)**	(972.36)	(858.82)	(821.92)	(762.46)	(1325.81)	(1810.00)
	95%	[-1140.47,	**[2398.46,**	[-20.74,	[-1107.04,	[-221.20,	[-784.28,	[-941.59,	[-696.96,
	CI	4667.86]	**5315.25]**	3845.24]	2306.94]	3056.55]	2291.03]	4384.35]	6608.48]
P&C $_t$	Est.	-1608.51	611.33	666.40	-171.42	-353.15	-1359.09	-38.32	-3845.79
	(SE)	(964.64)	(961.03)	(1013.67)	(929.60)	(744.44)	(991.88)	(1556.42)	(2531.98)
	95%	[-3528.20,	[-1301.93,	[-1348.71,	[-2019.11,	[-1837.53,	[-3359.41,	[-3164.49,	[-8955.54,
	CI	311.18]	2524.58]	2681.50]	1676.27]	1131.23]	641.23]	3087.85]	1263.95]
Commitment $_t$	Est.	-652.31	1115.01	933.82	1.30	-641.40	-1202.27	-1189.12	1014.20
	(SE)	(981.59)	(949.49)	(1047.47)	(945.30)	(737.08)	(963.47)	(1617.55)	(2247.09)
	95%	[-2605.74,	[-775.28,	[-1148.48,	[-1877.59,	[-2111.10,	[-3145.29,	[-4438.05,	[-3520.60,
	CI	1301.12]	3005.29]	3016.12]	1880.18]	828.29]	740.75]	2059.82]	5549.01]
P&C $_t$:	Est.	746.50	-811.69	-1826.03	1182.96	116.10	1890.07	-1087.87	2113.15
Commitment $_t$	(SE)	(1349.85)	(1353.80)	(1455.19)	(1345.24)	(1032.21)	(1402.44)	(2186.53)	(3593.89)
	95%	[-1939.79,	[-3506.90,	[-4718.86,	[-1490.85,	[-1942.08,	[-938.22,	[-5479.64,	[-5139.60,
	CI	3432.79]	1883.53]	1066.79]	3856.77]	2174.27]	4718.35]	3303.90]	9365.91]
Num.Obs.		88	86	94	95	79	51	58	50
R2		0.109	0.329	0.192	0.245	0.149	0.353	0.195	0.321
R2 Adj.		0.031	0.268	0.126	0.184	0.065	0.248	0.083	0.208
AIC		1672.4	1631.4	1803.3	1813.9	1451.0	948.7	1138.4	1011.1
BIC		1694.7	1653.5	1826.2	1836.9	1472.3	966.1	1157.0	1028.3
Log.Lik.		-827.217	-806.682	-892.641	-897.960	-716.479	-465.373	-560.222	-496.534
RMSE		2925.60	2867.18	3220.33	3081.80	2101.51	2221.70	3789.74	4972.85

Note. Participants are sorted in ascending order based on the number of missing values in the habit strength time series; Significant fixed effects (p-value $< .05$) are in bold; HS = habit strength; CBR = cue-behavior repetition; P&C = prompts and cues. Some participants exhibited very high occurrences of cue-behavior repetition (see Table S8), leading to limited variance in this variable and, ultimately, less accurate estimates of the effects.

Figure S7: N-of-1 Level Results for Model 3 – FOREST PLOT

N-of-1 Level Results for Model 3 (Dependent Variable is Steps) With the Target Operationalization of Cue-Behavior Repetition (CBR.1) for Participants With Less Than 20% Missing Values in the Habit Strength Time Series

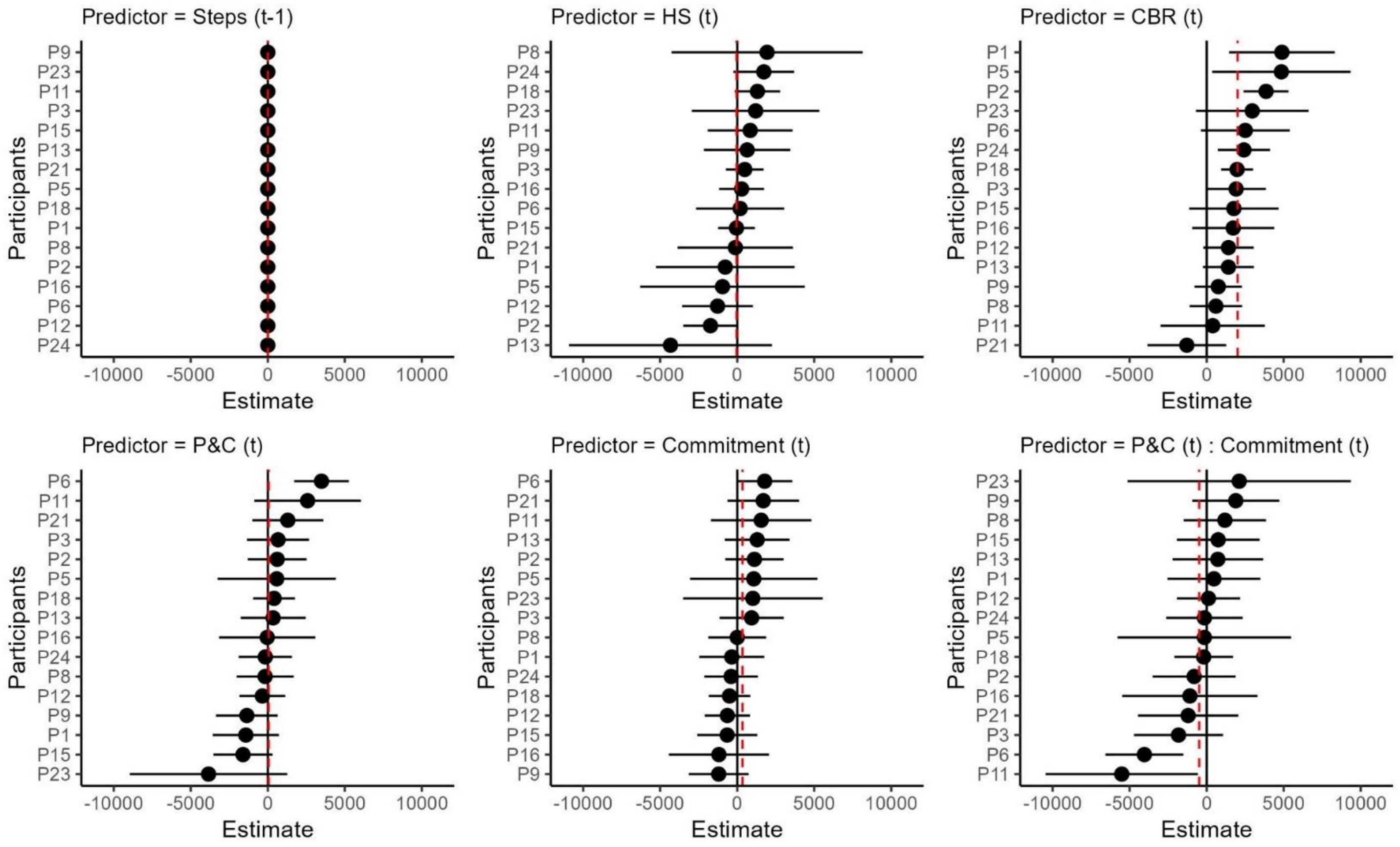

Note. The dashed red line represents the average estimate across participants. HS = habit strength; CBR = cue-behavior repetition; P&C = prompts and cues; (t) = the predictor measurement is taken on the same day as the dependent variable; (t-1) = the predictor measurement is taken on the previous day relative to the dependent variable. Some participants exhibited very high occurrences of cue-behavior repetition (see Table S8), leading to limited variance in this variable and, ultimately, less accurate estimates of the effects.

References

Gardner, B., Rebar, A. L., & Lally, P. (2022). How does habit form? Guidelines for tracking real-world habit formation. *Cogent Psychology*, *9*(1), 2041277. https://doi.org/10.1080/23311908.2022.2041277

Keller, J., Kwasnicka, D., Klaiber, P., Sichert, L., Lally, P., & Fleig, L. (2021a). Habit formation following routine-based versus time-based cue planning: A randomized controlled trial. *British Journal of Health Psychology*, *26*(3), 807–824. https://doi.org/10.1111/bjhp.12504

Keller, J., Kwasnicka, D., Klaiber, P., Sichert, L., Lally, P., & Fleig, L. (2021b). Habit formation following routine-based versus time-based cue planning: A randomized controlled trial. *British Journal of Health Psychology*, *26*(3), 807–824. https://doi.org/10.1111/bjhp.12504

Stadnitski, T., & Wild, B. (2019). How to Deal With Temporal Relationships Between Biopsychosocial Variables: A Practical Guide to Time Series Analysis. *Psychosomatic Medicine*, *81*(3), 289–304. https://doi.org/10.1097/PSY.0000000000000680

Tudor-Locke, C., Han, H., Aguiar, E. J., Barreira, T. V., Jr, J. M. S., Kang, M., & Rowe, D. A. (2018). How fast is fast enough? Walking cadence (steps/min) as a practical estimate of intensity in adults: a narrative review. *British Journal of Sports Medicine*, *52*(12), 776–788. https://doi.org/10.1136/bjsports-2017-097628
